# Supplementary material for: A Testosterone Metabolite 19-Hydroxyandrostenedione Induces Neuroendocrine Trans-Differentiation of Prostate Cancer Cells via an Ectopic Olfactory Receptor
Source: Front Oncol. 2018 May 28;8:162. doi: 10.3389/fonc.2018.00162 (PMC5985834; doi:10.3389/fonc.2018.00162)
Supplement: Supplementary file 1 [file presentation_1.pptx]

## Slide 1
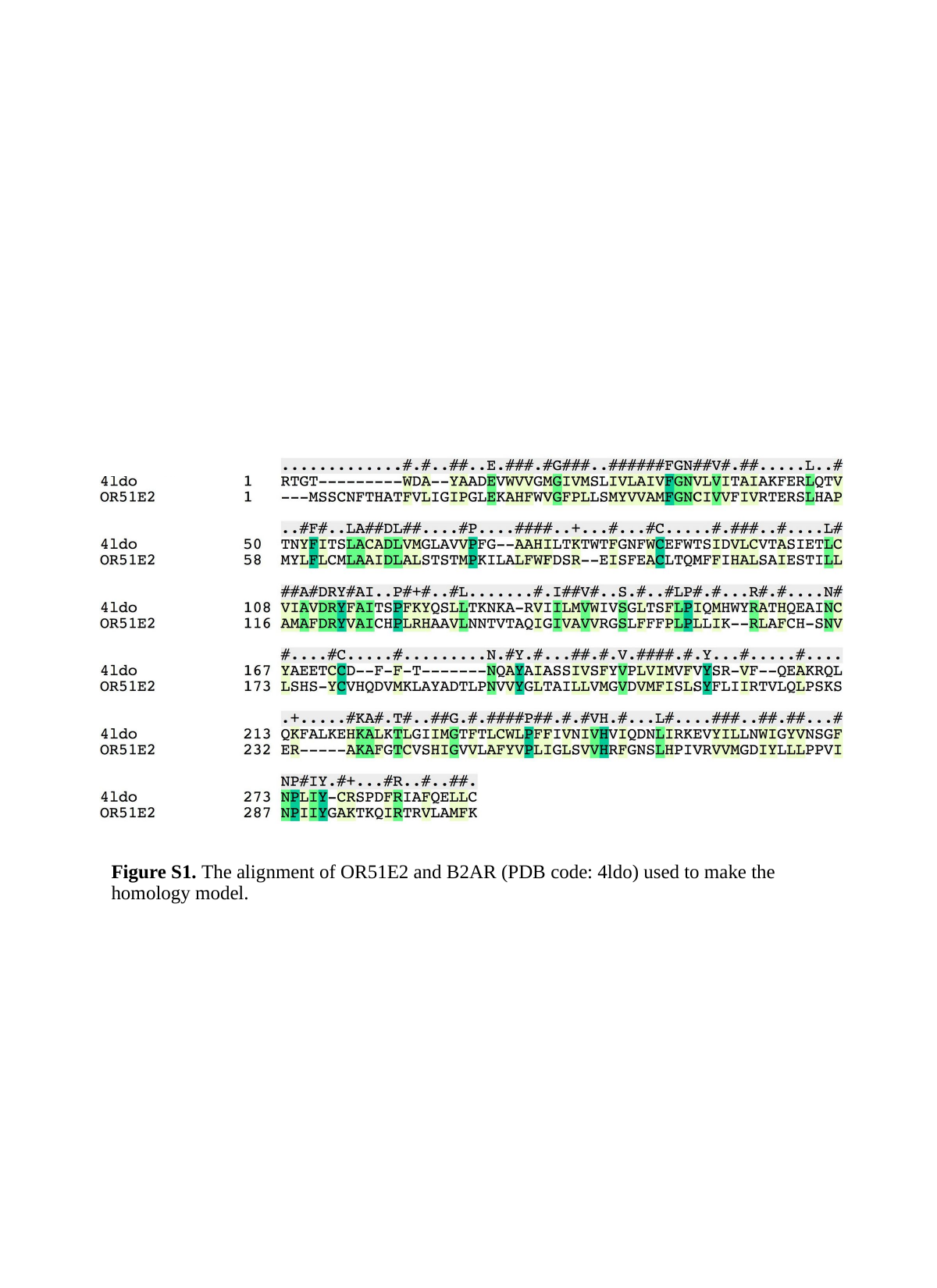

# Figure S1. The alignment of OR51E2 and B2AR (PDB code: 4ldo) used to make the homology model.

## Slide 2
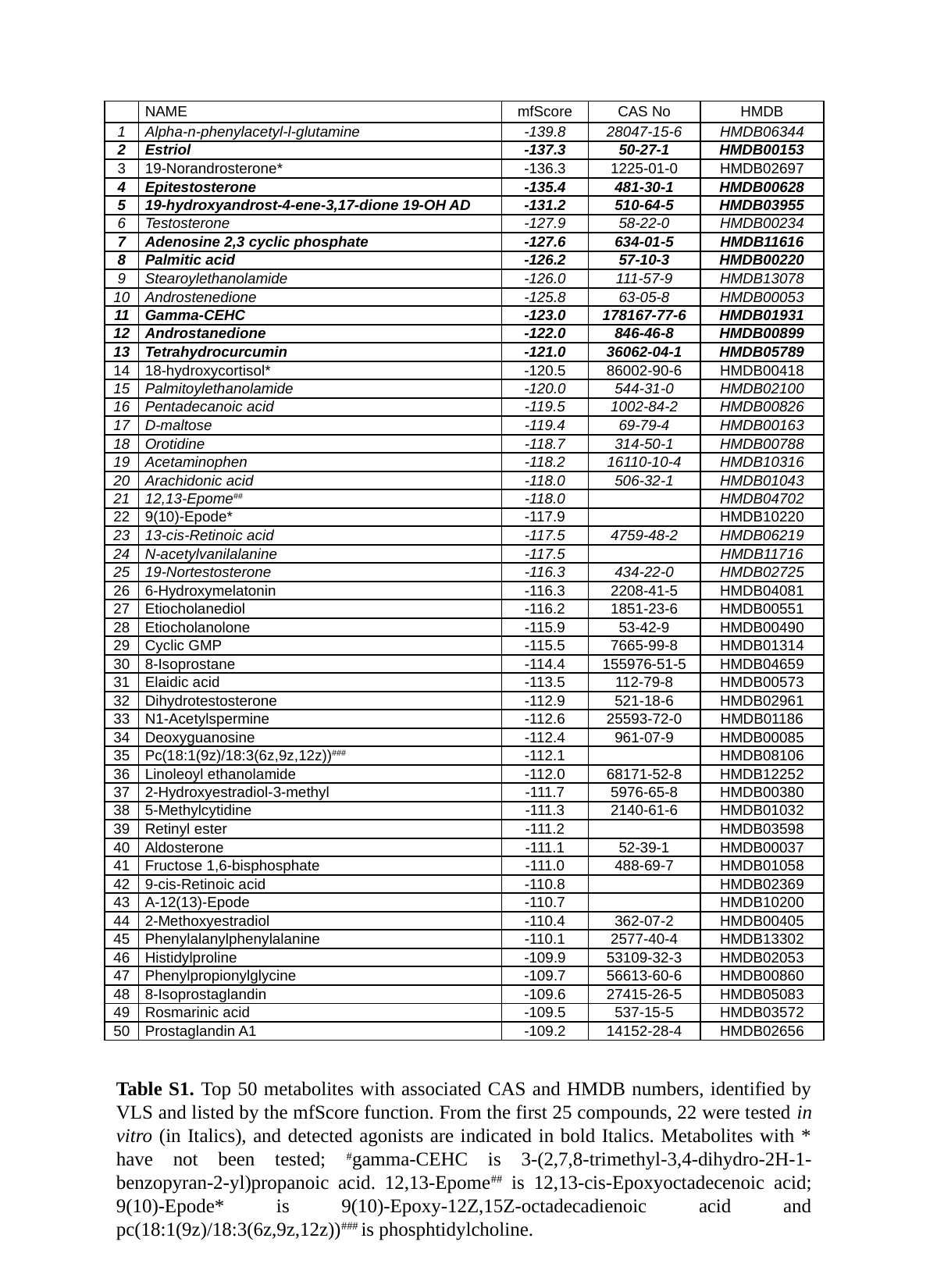

| | NAME | mfScore | CAS No | HMDB |
| --- | --- | --- | --- | --- |
| 1 | Alpha-n-phenylacetyl-l-glutamine | -139.8 | 28047-15-6 | HMDB06344 |
| 2 | Estriol | -137.3 | 50-27-1 | HMDB00153 |
| 3 | 19-Norandrosterone\* | -136.3 | 1225-01-0 | HMDB02697 |
| 4 | Epitestosterone | -135.4 | 481-30-1 | HMDB00628 |
| 5 | 19-hydroxyandrost-4-ene-3,17-dione 19-OH AD | -131.2 | 510-64-5 | HMDB03955 |
| 6 | Testosterone | -127.9 | 58-22-0 | HMDB00234 |
| 7 | Adenosine 2,3 cyclic phosphate | -127.6 | 634-01-5 | HMDB11616 |
| 8 | Palmitic acid | -126.2 | 57-10-3 | HMDB00220 |
| 9 | Stearoylethanolamide | -126.0 | 111-57-9 | HMDB13078 |
| 10 | Androstenedione | -125.8 | 63-05-8 | HMDB00053 |
| 11 | Gamma-CEHC | -123.0 | 178167-77-6 | HMDB01931 |
| 12 | Androstanedione | -122.0 | 846-46-8 | HMDB00899 |
| 13 | Tetrahydrocurcumin | -121.0 | 36062-04-1 | HMDB05789 |
| 14 | 18-hydroxycortisol\* | -120.5 | 86002-90-6 | HMDB00418 |
| 15 | Palmitoylethanolamide | -120.0 | 544-31-0 | HMDB02100 |
| 16 | Pentadecanoic acid | -119.5 | 1002-84-2 | HMDB00826 |
| 17 | D-maltose | -119.4 | 69-79-4 | HMDB00163 |
| 18 | Orotidine | -118.7 | 314-50-1 | HMDB00788 |
| 19 | Acetaminophen | -118.2 | 16110-10-4 | HMDB10316 |
| 20 | Arachidonic acid | -118.0 | 506-32-1 | HMDB01043 |
| 21 | 12,13-Epome## | -118.0 | | HMDB04702 |
| 22 | 9(10)-Epode\* | -117.9 | | HMDB10220 |
| 23 | 13-cis-Retinoic acid | -117.5 | 4759-48-2 | HMDB06219 |
| 24 | N-acetylvanilalanine | -117.5 | | HMDB11716 |
| 25 | 19-Nortestosterone | -116.3 | 434-22-0 | HMDB02725 |
| 26 | 6-Hydroxymelatonin | -116.3 | 2208-41-5 | HMDB04081 |
| 27 | Etiocholanediol | -116.2 | 1851-23-6 | HMDB00551 |
| 28 | Etiocholanolone | -115.9 | 53-42-9 | HMDB00490 |
| 29 | Cyclic GMP | -115.5 | 7665-99-8 | HMDB01314 |
| 30 | 8-Isoprostane | -114.4 | 155976-51-5 | HMDB04659 |
| 31 | Elaidic acid | -113.5 | 112-79-8 | HMDB00573 |
| 32 | Dihydrotestosterone | -112.9 | 521-18-6 | HMDB02961 |
| 33 | N1-Acetylspermine | -112.6 | 25593-72-0 | HMDB01186 |
| 34 | Deoxyguanosine | -112.4 | 961-07-9 | HMDB00085 |
| 35 | Pc(18:1(9z)/18:3(6z,9z,12z))### | -112.1 | | HMDB08106 |
| 36 | Linoleoyl ethanolamide | -112.0 | 68171-52-8 | HMDB12252 |
| 37 | 2-Hydroxyestradiol-3-methyl | -111.7 | 5976-65-8 | HMDB00380 |
| 38 | 5-Methylcytidine | -111.3 | 2140-61-6 | HMDB01032 |
| 39 | Retinyl ester | -111.2 | | HMDB03598 |
| 40 | Aldosterone | -111.1 | 52-39-1 | HMDB00037 |
| 41 | Fructose 1,6-bisphosphate | -111.0 | 488-69-7 | HMDB01058 |
| 42 | 9-cis-Retinoic acid | -110.8 | | HMDB02369 |
| 43 | A-12(13)-Epode | -110.7 | | HMDB10200 |
| 44 | 2-Methoxyestradiol | -110.4 | 362-07-2 | HMDB00405 |
| 45 | Phenylalanylphenylalanine | -110.1 | 2577-40-4 | HMDB13302 |
| 46 | Histidylproline | -109.9 | 53109-32-3 | HMDB02053 |
| 47 | Phenylpropionylglycine | -109.7 | 56613-60-6 | HMDB00860 |
| 48 | 8-Isoprostaglandin | -109.6 | 27415-26-5 | HMDB05083 |
| 49 | Rosmarinic acid | -109.5 | 537-15-5 | HMDB03572 |
| 50 | Prostaglandin A1 | -109.2 | 14152-28-4 | HMDB02656 |
Table S1. Top 50 metabolites with associated CAS and HMDB numbers, identified by VLS and listed by the mfScore function. From the first 25 compounds, 22 were tested in vitro (in Italics), and detected agonists are indicated in bold Italics. Metabolites with * have not been tested; #gamma-CEHC is 3-(2,7,8-trimethyl-3,4-dihydro-2H-1-benzopyran-2-yl)propanoic acid. 12,13-Epome## is 12,13-cis-Epoxyoctadecenoic acid; 9(10)-Epode* is 9(10)-Epoxy-12Z,15Z-octadecadienoic acid and pc(18:1(9z)/18:3(6z,9z,12z))### is phosphtidylcholine.

## Slide 3
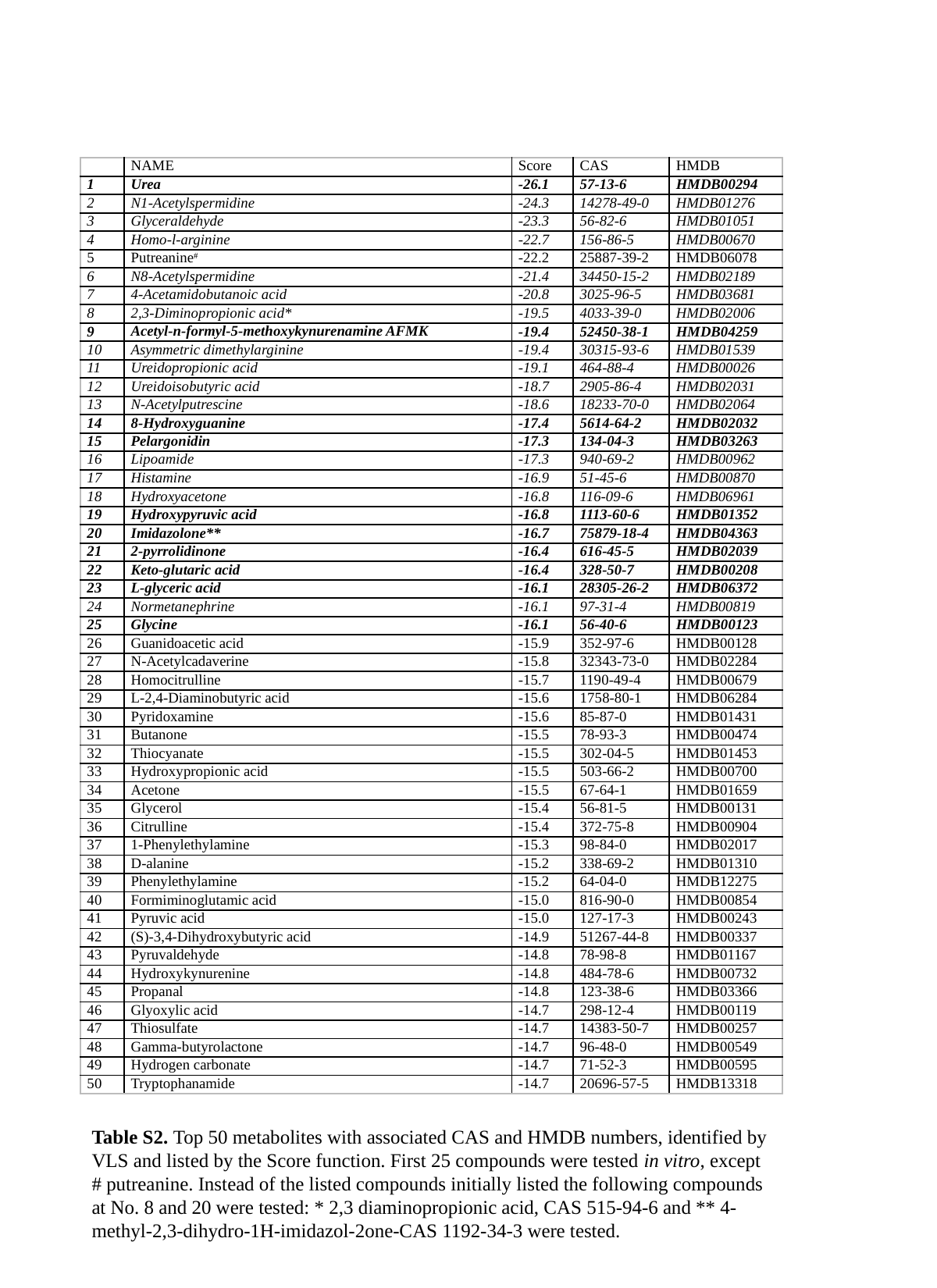

| | NAME | Score | CAS | HMDB |
| --- | --- | --- | --- | --- |
| 1 | Urea | -26.1 | 57-13-6 | HMDB00294 |
| 2 | N1-Acetylspermidine | -24.3 | 14278-49-0 | HMDB01276 |
| 3 | Glyceraldehyde | -23.3 | 56-82-6 | HMDB01051 |
| 4 | Homo-l-arginine | -22.7 | 156-86-5 | HMDB00670 |
| 5 | Putreanine# | -22.2 | 25887-39-2 | HMDB06078 |
| 6 | N8-Acetylspermidine | -21.4 | 34450-15-2 | HMDB02189 |
| 7 | 4-Acetamidobutanoic acid | -20.8 | 3025-96-5 | HMDB03681 |
| 8 | 2,3-Diminopropionic acid\* | -19.5 | 4033-39-0 | HMDB02006 |
| 9 | Acetyl-n-formyl-5-methoxykynurenamine AFMK | -19.4 | 52450-38-1 | HMDB04259 |
| 10 | Asymmetric dimethylarginine | -19.4 | 30315-93-6 | HMDB01539 |
| 11 | Ureidopropionic acid | -19.1 | 464-88-4 | HMDB00026 |
| 12 | Ureidoisobutyric acid | -18.7 | 2905-86-4 | HMDB02031 |
| 13 | N-Acetylputrescine | -18.6 | 18233-70-0 | HMDB02064 |
| 14 | 8-Hydroxyguanine | -17.4 | 5614-64-2 | HMDB02032 |
| 15 | Pelargonidin | -17.3 | 134-04-3 | HMDB03263 |
| 16 | Lipoamide | -17.3 | 940-69-2 | HMDB00962 |
| 17 | Histamine | -16.9 | 51-45-6 | HMDB00870 |
| 18 | Hydroxyacetone | -16.8 | 116-09-6 | HMDB06961 |
| 19 | Hydroxypyruvic acid | -16.8 | 1113-60-6 | HMDB01352 |
| 20 | Imidazolone\*\* | -16.7 | 75879-18-4 | HMDB04363 |
| 21 | 2-pyrrolidinone | -16.4 | 616-45-5 | HMDB02039 |
| 22 | Keto-glutaric acid | -16.4 | 328-50-7 | HMDB00208 |
| 23 | L-glyceric acid | -16.1 | 28305-26-2 | HMDB06372 |
| 24 | Normetanephrine | -16.1 | 97-31-4 | HMDB00819 |
| 25 | Glycine | -16.1 | 56-40-6 | HMDB00123 |
| 26 | Guanidoacetic acid | -15.9 | 352-97-6 | HMDB00128 |
| 27 | N-Acetylcadaverine | -15.8 | 32343-73-0 | HMDB02284 |
| 28 | Homocitrulline | -15.7 | 1190-49-4 | HMDB00679 |
| 29 | L-2,4-Diaminobutyric acid | -15.6 | 1758-80-1 | HMDB06284 |
| 30 | Pyridoxamine | -15.6 | 85-87-0 | HMDB01431 |
| 31 | Butanone | -15.5 | 78-93-3 | HMDB00474 |
| 32 | Thiocyanate | -15.5 | 302-04-5 | HMDB01453 |
| 33 | Hydroxypropionic acid | -15.5 | 503-66-2 | HMDB00700 |
| 34 | Acetone | -15.5 | 67-64-1 | HMDB01659 |
| 35 | Glycerol | -15.4 | 56-81-5 | HMDB00131 |
| 36 | Citrulline | -15.4 | 372-75-8 | HMDB00904 |
| 37 | 1-Phenylethylamine | -15.3 | 98-84-0 | HMDB02017 |
| 38 | D-alanine | -15.2 | 338-69-2 | HMDB01310 |
| 39 | Phenylethylamine | -15.2 | 64-04-0 | HMDB12275 |
| 40 | Formiminoglutamic acid | -15.0 | 816-90-0 | HMDB00854 |
| 41 | Pyruvic acid | -15.0 | 127-17-3 | HMDB00243 |
| 42 | (S)-3,4-Dihydroxybutyric acid | -14.9 | 51267-44-8 | HMDB00337 |
| 43 | Pyruvaldehyde | -14.8 | 78-98-8 | HMDB01167 |
| 44 | Hydroxykynurenine | -14.8 | 484-78-6 | HMDB00732 |
| 45 | Propanal | -14.8 | 123-38-6 | HMDB03366 |
| 46 | Glyoxylic acid | -14.7 | 298-12-4 | HMDB00119 |
| 47 | Thiosulfate | -14.7 | 14383-50-7 | HMDB00257 |
| 48 | Gamma-butyrolactone | -14.7 | 96-48-0 | HMDB00549 |
| 49 | Hydrogen carbonate | -14.7 | 71-52-3 | HMDB00595 |
| 50 | Tryptophanamide | -14.7 | 20696-57-5 | HMDB13318 |
Table S2. Top 50 metabolites with associated CAS and HMDB numbers, identified by VLS and listed by the Score function. First 25 compounds were tested in vitro, except # putreanine. Instead of the listed compounds initially listed the following compounds at No. 8 and 20 were tested: * 2,3 diaminopropionic acid, CAS 515-94-6 and ** 4-methyl-2,3-dihydro-1H-imidazol-2one-CAS 1192-34-3 were tested.

## Slide 4
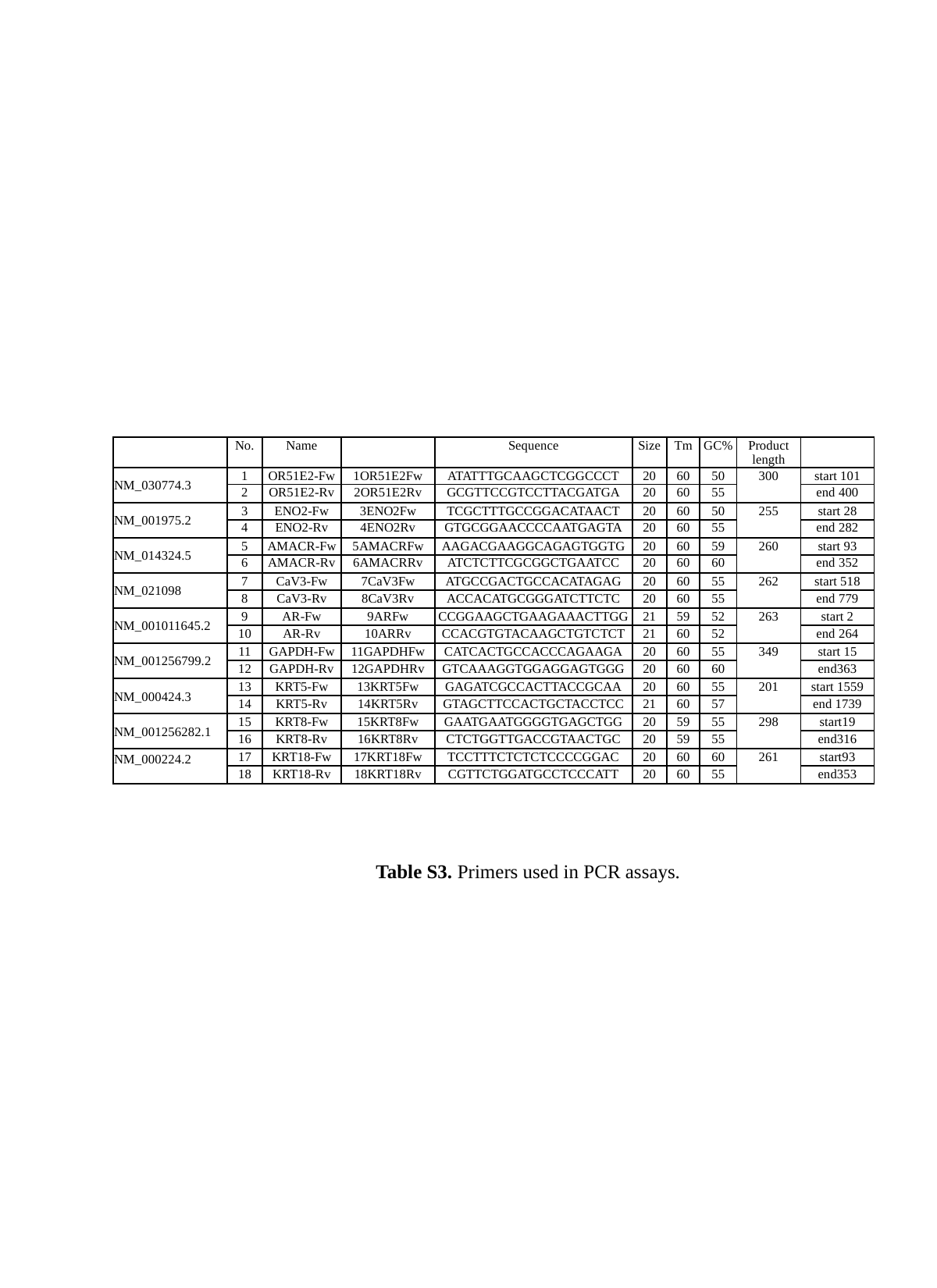

| | No. | Name | | Sequence | Size | Tm | GC% | Product length | |
| --- | --- | --- | --- | --- | --- | --- | --- | --- | --- |
| NM\_030774.3 | 1 | OR51E2-Fw | 1OR51E2Fw | ATATTTGCAAGCTCGGCCCT | 20 | 60 | 50 | 300 | start 101 |
| | 2 | OR51E2-Rv | 2OR51E2Rv | GCGTTCCGTCCTTACGATGA | 20 | 60 | 55 | | end 400 |
| NM\_001975.2 | 3 | ENO2-Fw | 3ENO2Fw | TCGCTTTGCCGGACATAACT | 20 | 60 | 50 | 255 | start 28 |
| | 4 | ENO2-Rv | 4ENO2Rv | GTGCGGAACCCCAATGAGTA | 20 | 60 | 55 | | end 282 |
| NM\_014324.5 | 5 | AMACR-Fw | 5AMACRFw | AAGACGAAGGCAGAGTGGTG | 20 | 60 | 59 | 260 | start 93 |
| | 6 | AMACR-Rv | 6AMACRRv | ATCTCTTCGCGGCTGAATCC | 20 | 60 | 60 | | end 352 |
| NM\_021098 | 7 | CaV3-Fw | 7CaV3Fw | ATGCCGACTGCCACATAGAG | 20 | 60 | 55 | 262 | start 518 |
| | 8 | CaV3-Rv | 8CaV3Rv | ACCACATGCGGGATCTTCTC | 20 | 60 | 55 | | end 779 |
| NM\_001011645.2 | 9 | AR-Fw | 9ARFw | CCGGAAGCTGAAGAAACTTGG | 21 | 59 | 52 | 263 | start 2 |
| | 10 | AR-Rv | 10ARRv | CCACGTGTACAAGCTGTCTCT | 21 | 60 | 52 | | end 264 |
| NM\_001256799.2 | 11 | GAPDH-Fw | 11GAPDHFw | CATCACTGCCACCCAGAAGA | 20 | 60 | 55 | 349 | start 15 |
| | 12 | GAPDH-Rv | 12GAPDHRv | GTCAAAGGTGGAGGAGTGGG | 20 | 60 | 60 | | end363 |
| NM\_000424.3 | 13 | KRT5-Fw | 13KRT5Fw | GAGATCGCCACTTACCGCAA | 20 | 60 | 55 | 201 | start 1559 |
| | 14 | KRT5-Rv | 14KRT5Rv | GTAGCTTCCACTGCTACCTCC | 21 | 60 | 57 | | end 1739 |
| NM\_001256282.1 | 15 | KRT8-Fw | 15KRT8Fw | GAATGAATGGGGTGAGCTGG | 20 | 59 | 55 | 298 | start19 |
| | 16 | KRT8-Rv | 16KRT8Rv | CTCTGGTTGACCGTAACTGC | 20 | 59 | 55 | | end316 |
| NM\_000224.2 | 17 | KRT18-Fw | 17KRT18Fw | TCCTTTCTCTCTCCCCGGAC | 20 | 60 | 60 | 261 | start93 |
| | 18 | KRT18-Rv | 18KRT18Rv | CGTTCTGGATGCCTCCCATT | 20 | 60 | 55 | | end353 |
Table S3. Primers used in PCR assays.

## Slide 5
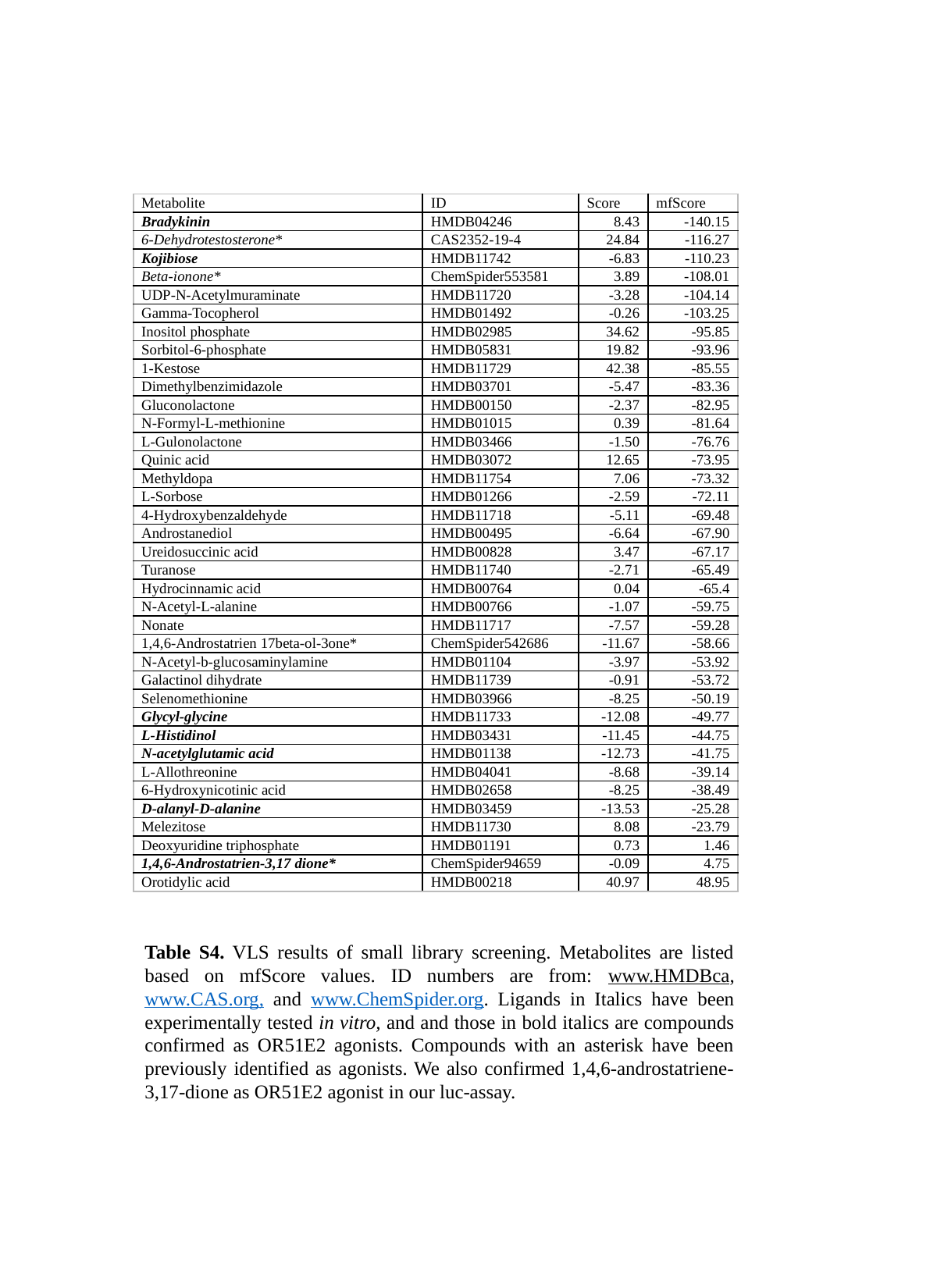

| Metabolite | ID | Score | mfScore |
| --- | --- | --- | --- |
| Bradykinin | HMDB04246 | 8.43 | -140.15 |
| 6-Dehydrotestosterone\* | CAS2352-19-4 | 24.84 | -116.27 |
| Kojibiose | HMDB11742 | -6.83 | -110.23 |
| Beta-ionone\* | ChemSpider553581 | 3.89 | -108.01 |
| UDP-N-Acetylmuraminate | HMDB11720 | -3.28 | -104.14 |
| Gamma-Tocopherol | HMDB01492 | -0.26 | -103.25 |
| Inositol phosphate | HMDB02985 | 34.62 | -95.85 |
| Sorbitol-6-phosphate | HMDB05831 | 19.82 | -93.96 |
| 1-Kestose | HMDB11729 | 42.38 | -85.55 |
| Dimethylbenzimidazole | HMDB03701 | -5.47 | -83.36 |
| Gluconolactone | HMDB00150 | -2.37 | -82.95 |
| N-Formyl-L-methionine | HMDB01015 | 0.39 | -81.64 |
| L-Gulonolactone | HMDB03466 | -1.50 | -76.76 |
| Quinic acid | HMDB03072 | 12.65 | -73.95 |
| Methyldopa | HMDB11754 | 7.06 | -73.32 |
| L-Sorbose | HMDB01266 | -2.59 | -72.11 |
| 4-Hydroxybenzaldehyde | HMDB11718 | -5.11 | -69.48 |
| Androstanediol | HMDB00495 | -6.64 | -67.90 |
| Ureidosuccinic acid | HMDB00828 | 3.47 | -67.17 |
| Turanose | HMDB11740 | -2.71 | -65.49 |
| Hydrocinnamic acid | HMDB00764 | 0.04 | -65.4 |
| N-Acetyl-L-alanine | HMDB00766 | -1.07 | -59.75 |
| Nonate | HMDB11717 | -7.57 | -59.28 |
| 1,4,6-Androstatrien 17beta-ol-3one\* | ChemSpider542686 | -11.67 | -58.66 |
| N-Acetyl-b-glucosaminylamine | HMDB01104 | -3.97 | -53.92 |
| Galactinol dihydrate | HMDB11739 | -0.91 | -53.72 |
| Selenomethionine | HMDB03966 | -8.25 | -50.19 |
| Glycyl-glycine | HMDB11733 | -12.08 | -49.77 |
| L-Histidinol | HMDB03431 | -11.45 | -44.75 |
| N-acetylglutamic acid | HMDB01138 | -12.73 | -41.75 |
| L-Allothreonine | HMDB04041 | -8.68 | -39.14 |
| 6-Hydroxynicotinic acid | HMDB02658 | -8.25 | -38.49 |
| D-alanyl-D-alanine | HMDB03459 | -13.53 | -25.28 |
| Melezitose | HMDB11730 | 8.08 | -23.79 |
| Deoxyuridine triphosphate | HMDB01191 | 0.73 | 1.46 |
| 1,4,6-Androstatrien-3,17 dione\* | ChemSpider94659 | -0.09 | 4.75 |
| Orotidylic acid | HMDB00218 | 40.97 | 48.95 |
Table S4. VLS results of small library screening. Metabolites are listed based on mfScore values. ID numbers are from: www.HMDBca, www.CAS.org, and www.ChemSpider.org. Ligands in Italics have been experimentally tested in vitro, and and those in bold italics are compounds confirmed as OR51E2 agonists. Compounds with an asterisk have been previously identified as agonists. We also confirmed 1,4,6-androstatriene-3,17-dione as OR51E2 agonist in our luc-assay.

## Slide 6
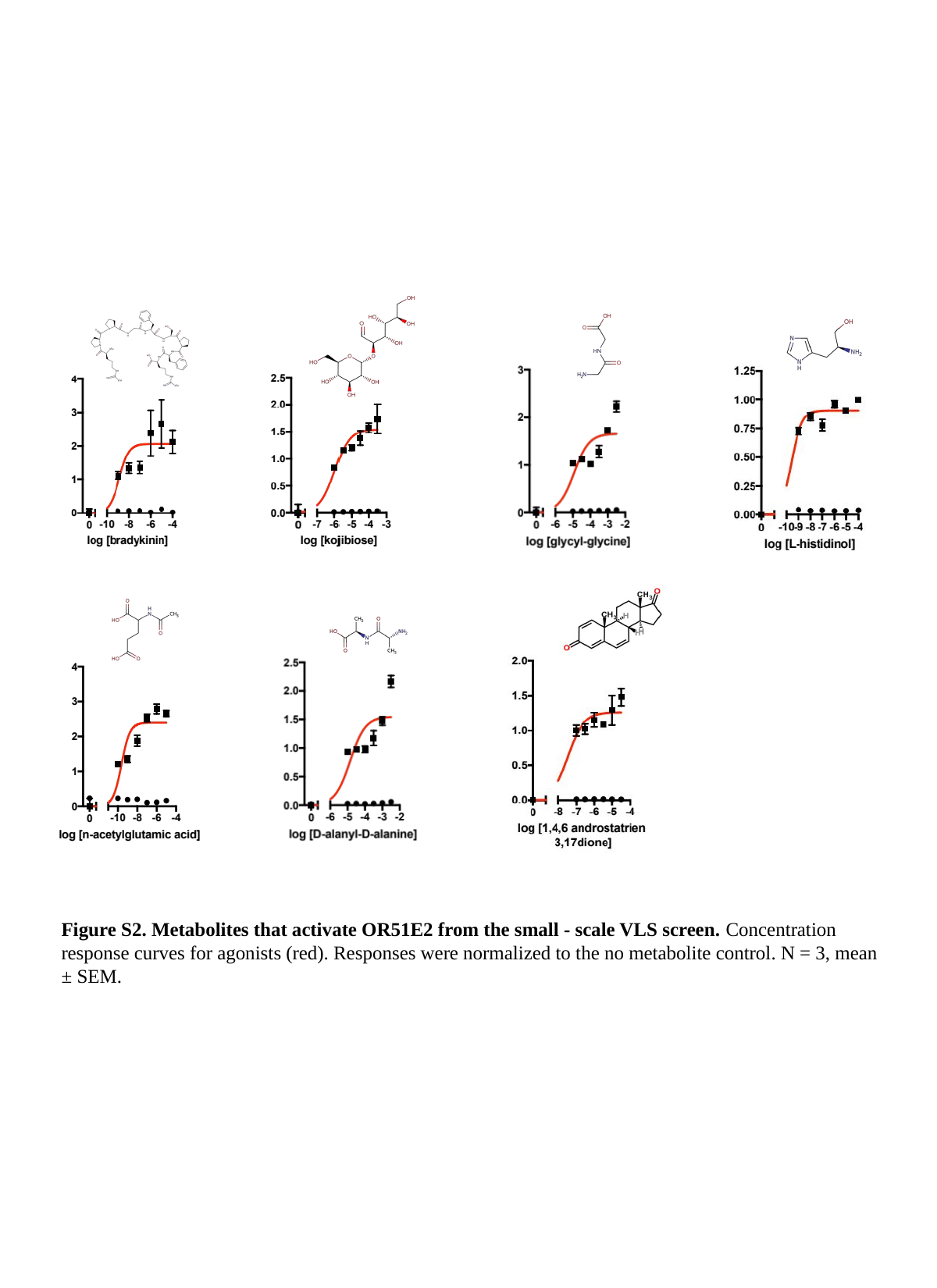

Figure S2. Metabolites that activate OR51E2 from the small - scale VLS screen. Concentration response curves for agonists (red). Responses were normalized to the no metabolite control. N = 3, mean ± SEM.

## Slide 7
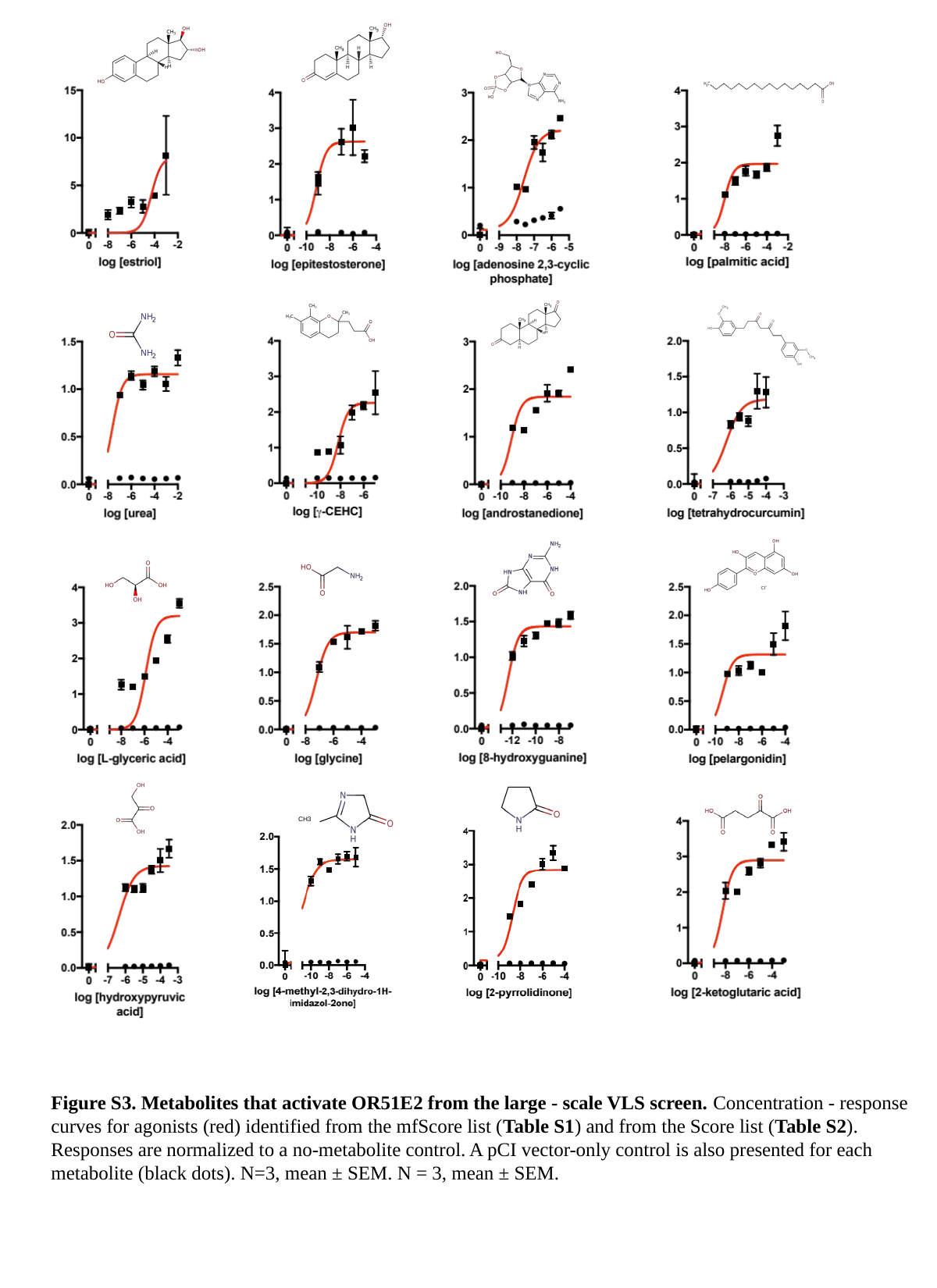

CH3
Figure S3. Metabolites that activate OR51E2 from the large - scale VLS screen. Concentration - response curves for agonists (red) identified from the mfScore list (Table S1) and from the Score list (Table S2). Responses are normalized to a no-metabolite control. A pCI vector-only control is also presented for each metabolite (black dots). N=3, mean ± SEM. N = 3, mean ± SEM.

## Slide 8
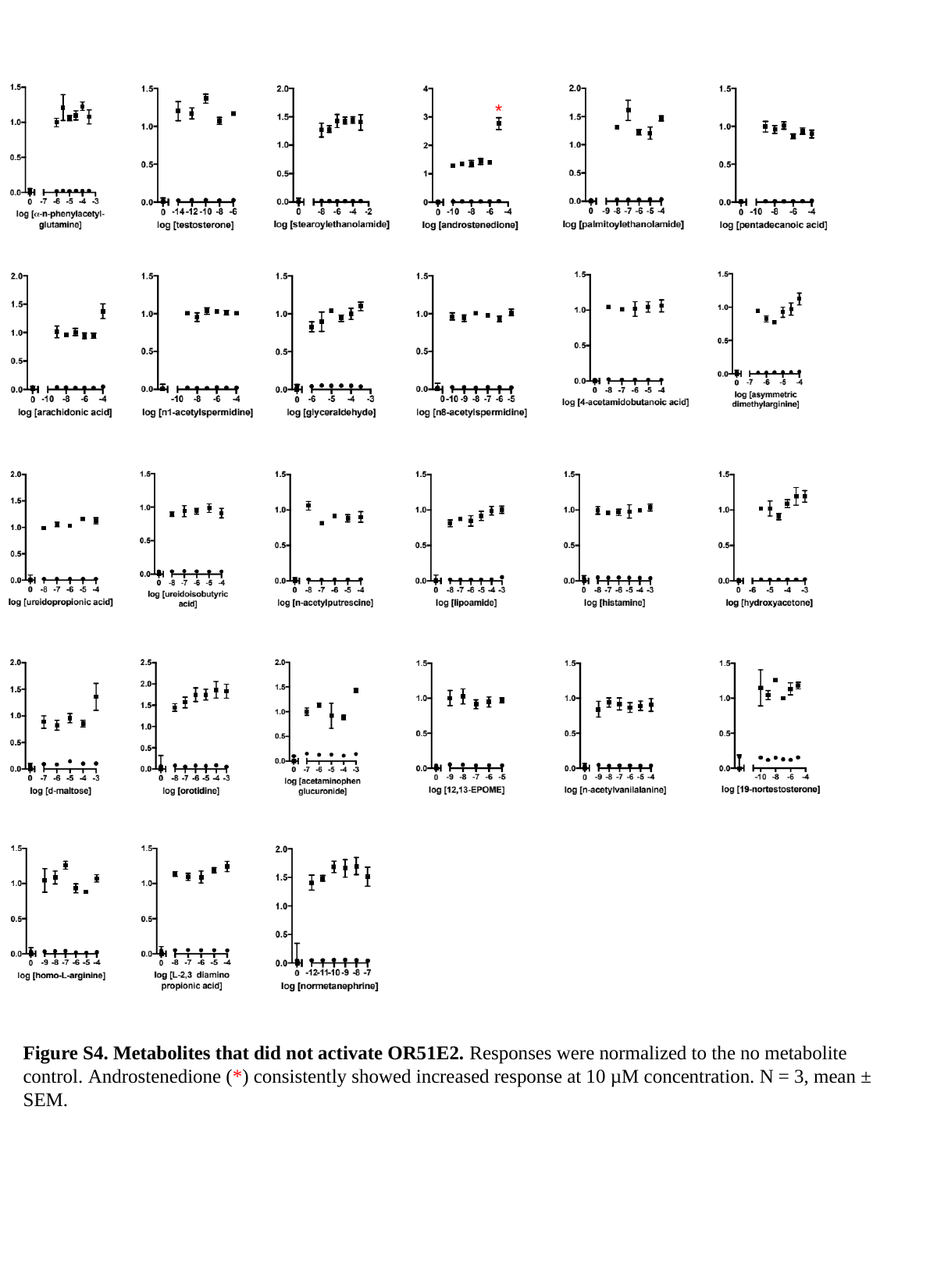

*
Figure S4. Metabolites that did not activate OR51E2. Responses were normalized to the no metabolite control. Androstenedione (*) consistently showed increased response at 10 µM concentration. N = 3, mean ± SEM.

## Slide 9
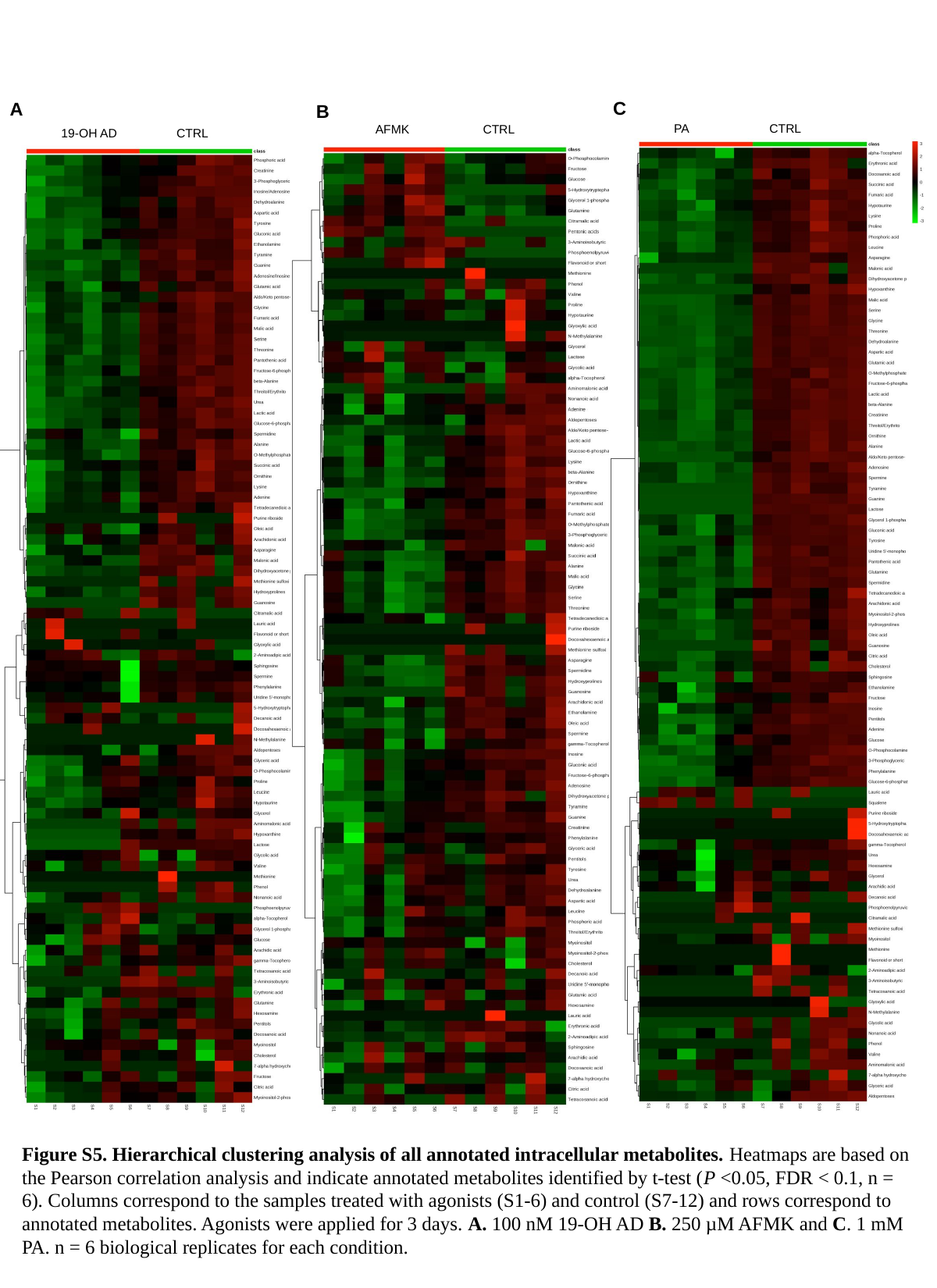

C
A
B
PA CTRL
AFMK CTRL
19-OH AD CTRL
Figure S5. Hierarchical clustering analysis of all annotated intracellular metabolites. Heatmaps are based on the Pearson correlation analysis and indicate annotated metabolites identified by t-test (P <0.05, FDR < 0.1, n = 6). Columns correspond to the samples treated with agonists (S1-6) and control (S7-12) and rows correspond to annotated metabolites. Agonists were applied for 3 days. A. 100 nM 19-OH AD B. 250 µM AFMK and C. 1 mM PA. n = 6 biological replicates for each condition.

## Slide 10
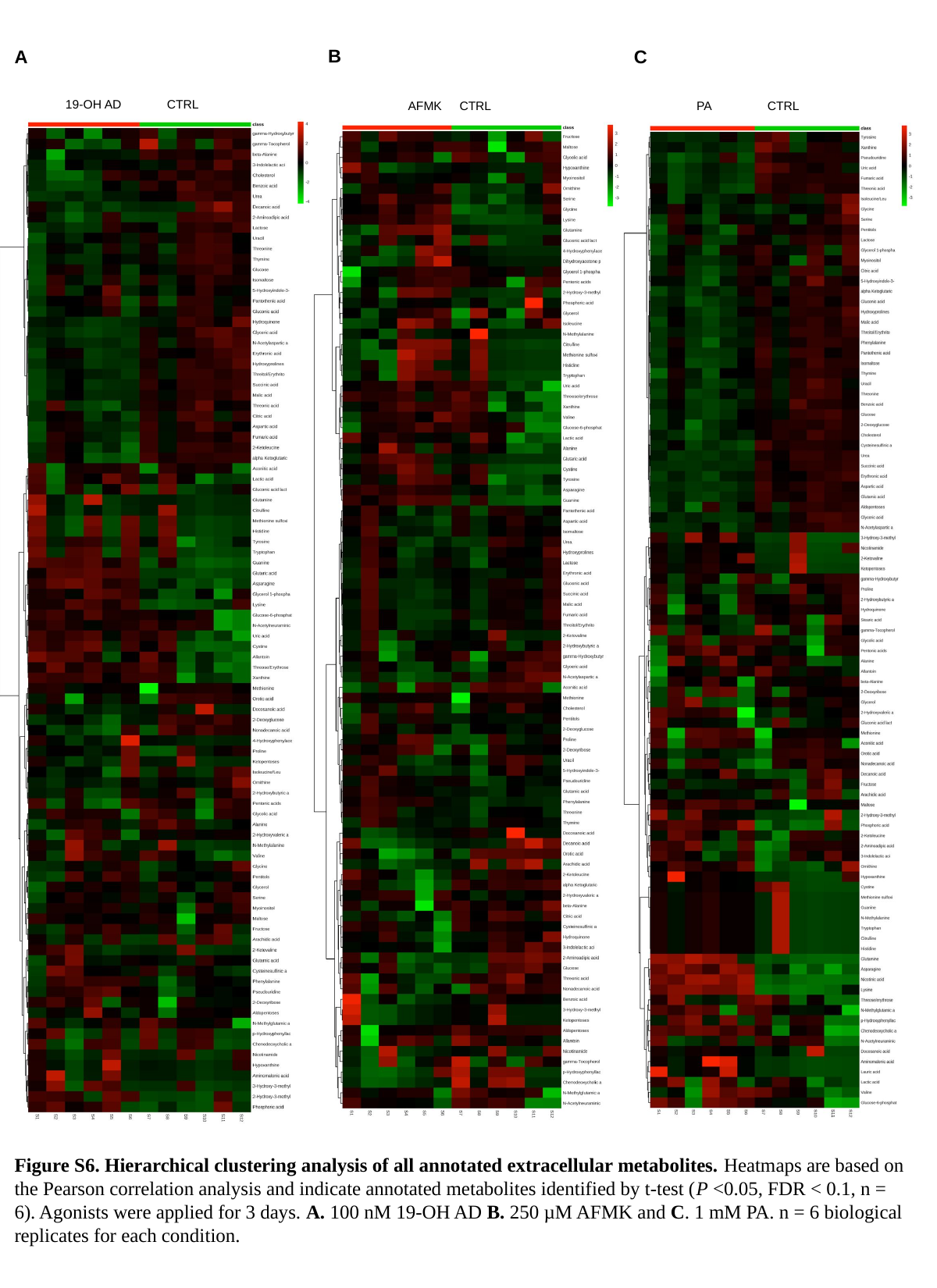

B
A
C
19-OH AD CTRL
AFMK CTRL
PA CTRL
Figure S6. Hierarchical clustering analysis of all annotated extracellular metabolites. Heatmaps are based on the Pearson correlation analysis and indicate annotated metabolites identified by t-test (P <0.05, FDR < 0.1, n = 6). Agonists were applied for 3 days. A. 100 nM 19-OH AD B. 250 µM AFMK and C. 1 mM PA. n = 6 biological replicates for each condition.

## Slide 11
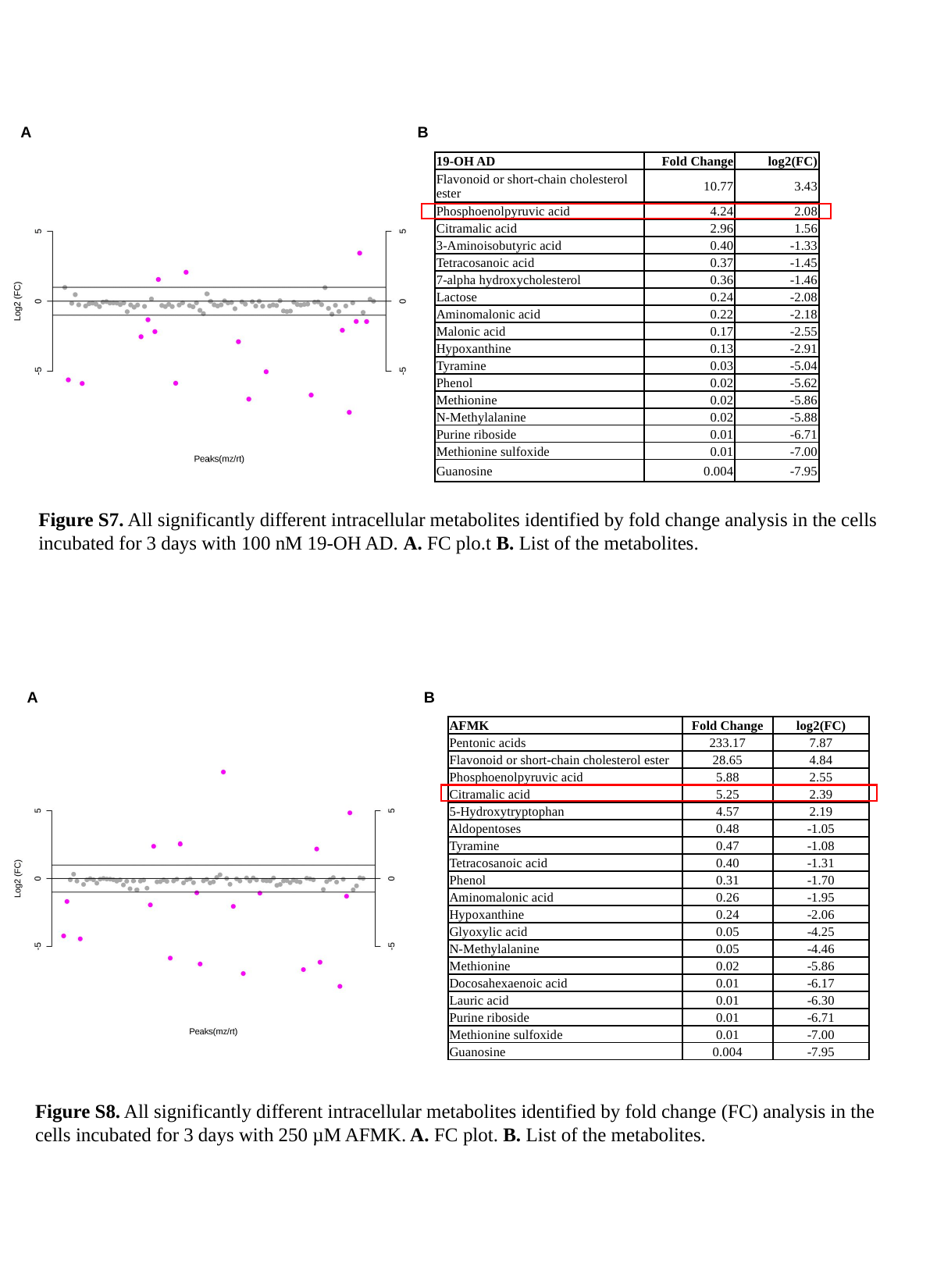

A B
| 19-OH AD | Fold Change | log2(FC) |
| --- | --- | --- |
| Flavonoid or short-chain cholesterol ester | 10.77 | 3.43 |
| Phosphoenolpyruvic acid | 4.24 | 2.08 |
| Citramalic acid | 2.96 | 1.56 |
| 3-Aminoisobutyric acid | 0.40 | -1.33 |
| Tetracosanoic acid | 0.37 | -1.45 |
| 7-alpha hydroxycholesterol | 0.36 | -1.46 |
| Lactose | 0.24 | -2.08 |
| Aminomalonic acid | 0.22 | -2.18 |
| Malonic acid | 0.17 | -2.55 |
| Hypoxanthine | 0.13 | -2.91 |
| Tyramine | 0.03 | -5.04 |
| Phenol | 0.02 | -5.62 |
| Methionine | 0.02 | -5.86 |
| N-Methylalanine | 0.02 | -5.88 |
| Purine riboside | 0.01 | -6.71 |
| Methionine sulfoxide | 0.01 | -7.00 |
| Guanosine | 0.004 | -7.95 |
Figure S7. All significantly different intracellular metabolites identified by fold change analysis in the cells incubated for 3 days with 100 nM 19-OH AD. A. FC plo.t B. List of the metabolites.
A B
| AFMK | Fold Change | log2(FC) |
| --- | --- | --- |
| Pentonic acids | 233.17 | 7.87 |
| Flavonoid or short-chain cholesterol ester | 28.65 | 4.84 |
| Phosphoenolpyruvic acid | 5.88 | 2.55 |
| Citramalic acid | 5.25 | 2.39 |
| 5-Hydroxytryptophan | 4.57 | 2.19 |
| Aldopentoses | 0.48 | -1.05 |
| Tyramine | 0.47 | -1.08 |
| Tetracosanoic acid | 0.40 | -1.31 |
| Phenol | 0.31 | -1.70 |
| Aminomalonic acid | 0.26 | -1.95 |
| Hypoxanthine | 0.24 | -2.06 |
| Glyoxylic acid | 0.05 | -4.25 |
| N-Methylalanine | 0.05 | -4.46 |
| Methionine | 0.02 | -5.86 |
| Docosahexaenoic acid | 0.01 | -6.17 |
| Lauric acid | 0.01 | -6.30 |
| Purine riboside | 0.01 | -6.71 |
| Methionine sulfoxide | 0.01 | -7.00 |
| Guanosine | 0.004 | -7.95 |
Figure S8. All significantly different intracellular metabolites identified by fold change (FC) analysis in the cells incubated for 3 days with 250 µM AFMK. A. FC plot. B. List of the metabolites.

## Slide 12
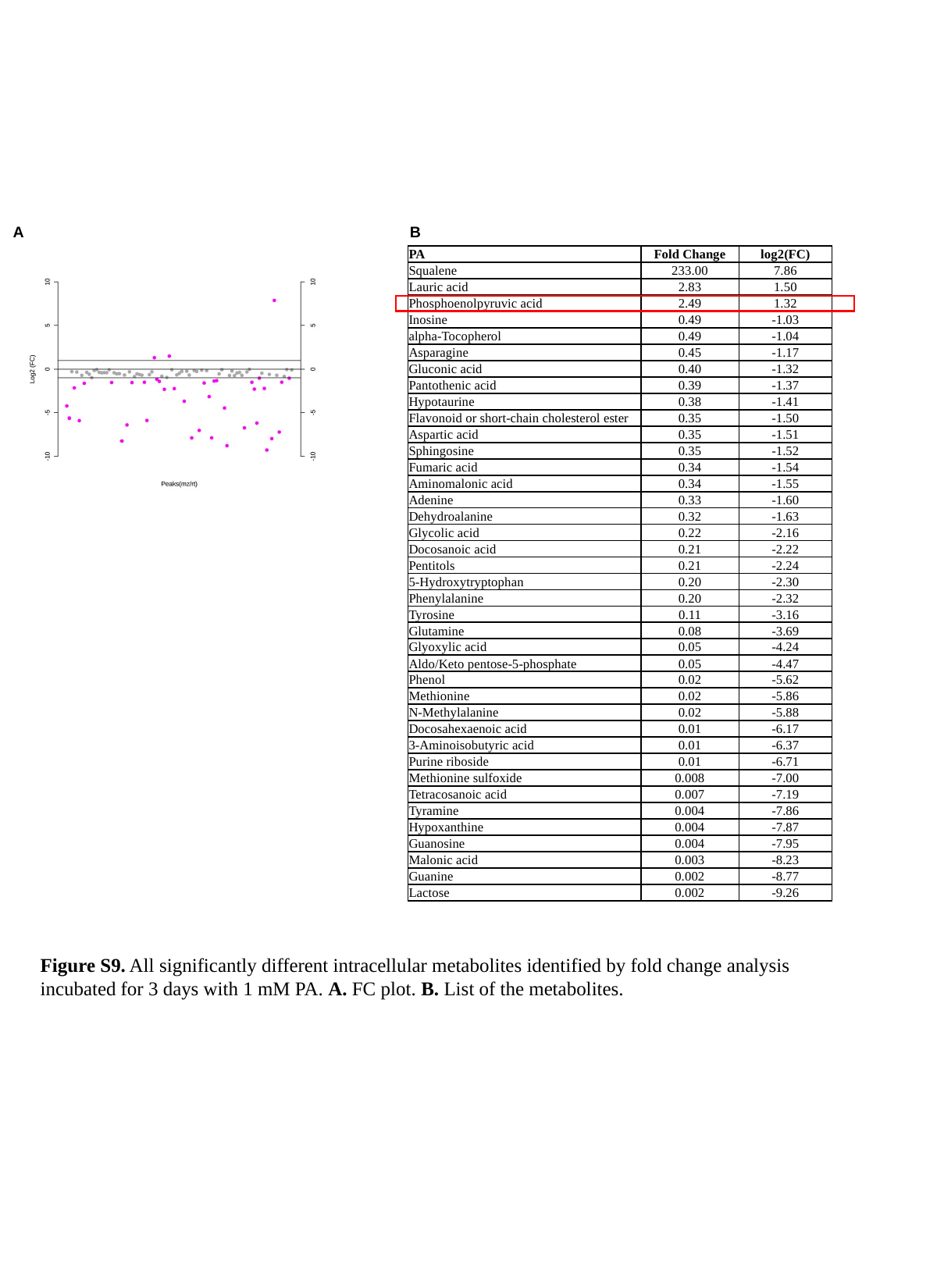

A B
| PA | Fold Change | log2(FC) |
| --- | --- | --- |
| Squalene | 233.00 | 7.86 |
| Lauric acid | 2.83 | 1.50 |
| Phosphoenolpyruvic acid | 2.49 | 1.32 |
| Inosine | 0.49 | -1.03 |
| alpha-Tocopherol | 0.49 | -1.04 |
| Asparagine | 0.45 | -1.17 |
| Gluconic acid | 0.40 | -1.32 |
| Pantothenic acid | 0.39 | -1.37 |
| Hypotaurine | 0.38 | -1.41 |
| Flavonoid or short-chain cholesterol ester | 0.35 | -1.50 |
| Aspartic acid | 0.35 | -1.51 |
| Sphingosine | 0.35 | -1.52 |
| Fumaric acid | 0.34 | -1.54 |
| Aminomalonic acid | 0.34 | -1.55 |
| Adenine | 0.33 | -1.60 |
| Dehydroalanine | 0.32 | -1.63 |
| Glycolic acid | 0.22 | -2.16 |
| Docosanoic acid | 0.21 | -2.22 |
| Pentitols | 0.21 | -2.24 |
| 5-Hydroxytryptophan | 0.20 | -2.30 |
| Phenylalanine | 0.20 | -2.32 |
| Tyrosine | 0.11 | -3.16 |
| Glutamine | 0.08 | -3.69 |
| Glyoxylic acid | 0.05 | -4.24 |
| Aldo/Keto pentose-5-phosphate | 0.05 | -4.47 |
| Phenol | 0.02 | -5.62 |
| Methionine | 0.02 | -5.86 |
| N-Methylalanine | 0.02 | -5.88 |
| Docosahexaenoic acid | 0.01 | -6.17 |
| 3-Aminoisobutyric acid | 0.01 | -6.37 |
| Purine riboside | 0.01 | -6.71 |
| Methionine sulfoxide | 0.008 | -7.00 |
| Tetracosanoic acid | 0.007 | -7.19 |
| Tyramine | 0.004 | -7.86 |
| Hypoxanthine | 0.004 | -7.87 |
| Guanosine | 0.004 | -7.95 |
| Malonic acid | 0.003 | -8.23 |
| Guanine | 0.002 | -8.77 |
| Lactose | 0.002 | -9.26 |
Figure S9. All significantly different intracellular metabolites identified by fold change analysis incubated for 3 days with 1 mM PA. A. FC plot. B. List of the metabolites.

## Slide 13
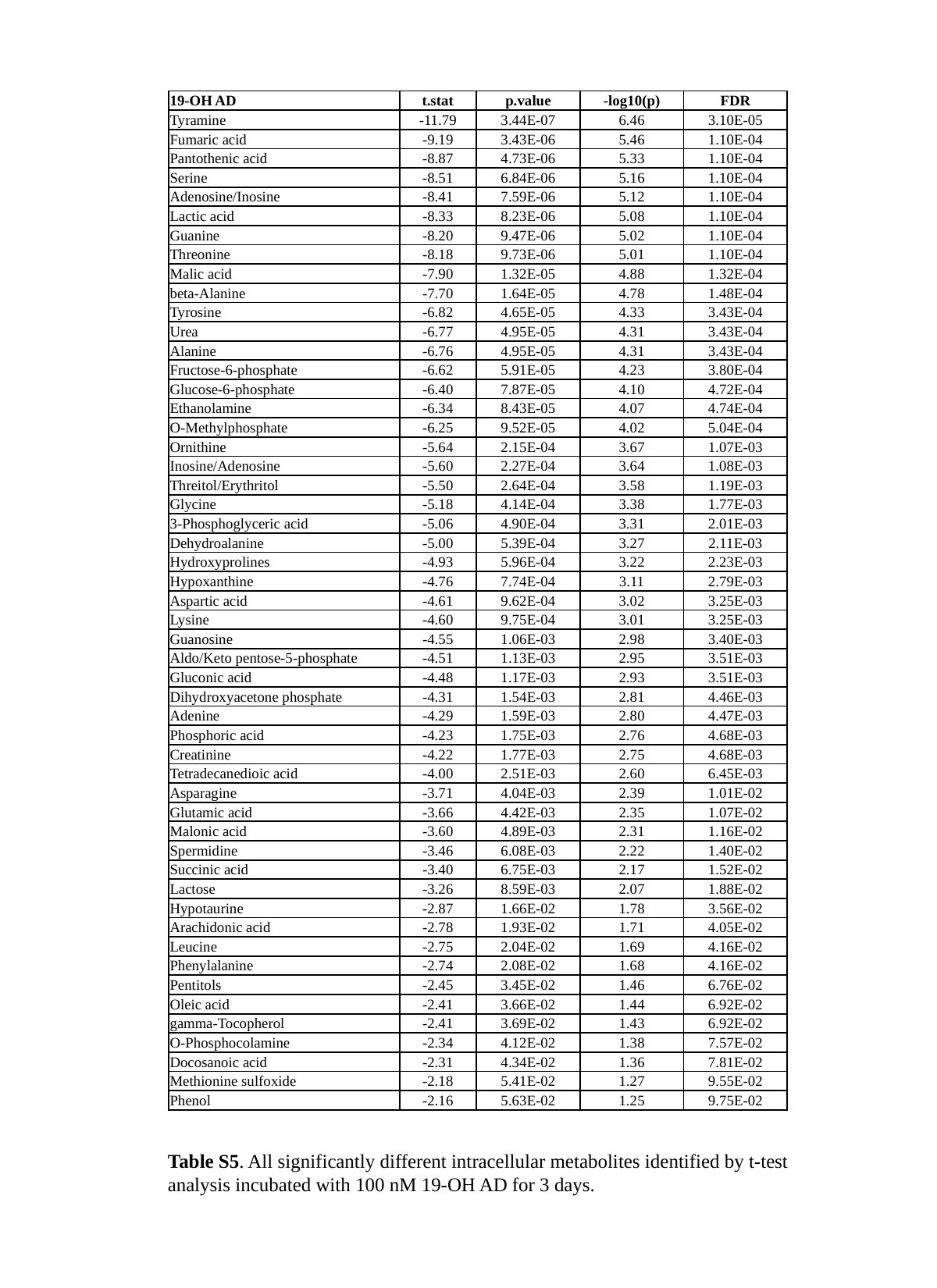

| 19-OH AD | t.stat | p.value | -log10(p) | FDR |
| --- | --- | --- | --- | --- |
| Tyramine | -11.79 | 3.44E-07 | 6.46 | 3.10E-05 |
| Fumaric acid | -9.19 | 3.43E-06 | 5.46 | 1.10E-04 |
| Pantothenic acid | -8.87 | 4.73E-06 | 5.33 | 1.10E-04 |
| Serine | -8.51 | 6.84E-06 | 5.16 | 1.10E-04 |
| Adenosine/Inosine | -8.41 | 7.59E-06 | 5.12 | 1.10E-04 |
| Lactic acid | -8.33 | 8.23E-06 | 5.08 | 1.10E-04 |
| Guanine | -8.20 | 9.47E-06 | 5.02 | 1.10E-04 |
| Threonine | -8.18 | 9.73E-06 | 5.01 | 1.10E-04 |
| Malic acid | -7.90 | 1.32E-05 | 4.88 | 1.32E-04 |
| beta-Alanine | -7.70 | 1.64E-05 | 4.78 | 1.48E-04 |
| Tyrosine | -6.82 | 4.65E-05 | 4.33 | 3.43E-04 |
| Urea | -6.77 | 4.95E-05 | 4.31 | 3.43E-04 |
| Alanine | -6.76 | 4.95E-05 | 4.31 | 3.43E-04 |
| Fructose-6-phosphate | -6.62 | 5.91E-05 | 4.23 | 3.80E-04 |
| Glucose-6-phosphate | -6.40 | 7.87E-05 | 4.10 | 4.72E-04 |
| Ethanolamine | -6.34 | 8.43E-05 | 4.07 | 4.74E-04 |
| O-Methylphosphate | -6.25 | 9.52E-05 | 4.02 | 5.04E-04 |
| Ornithine | -5.64 | 2.15E-04 | 3.67 | 1.07E-03 |
| Inosine/Adenosine | -5.60 | 2.27E-04 | 3.64 | 1.08E-03 |
| Threitol/Erythritol | -5.50 | 2.64E-04 | 3.58 | 1.19E-03 |
| Glycine | -5.18 | 4.14E-04 | 3.38 | 1.77E-03 |
| 3-Phosphoglyceric acid | -5.06 | 4.90E-04 | 3.31 | 2.01E-03 |
| Dehydroalanine | -5.00 | 5.39E-04 | 3.27 | 2.11E-03 |
| Hydroxyprolines | -4.93 | 5.96E-04 | 3.22 | 2.23E-03 |
| Hypoxanthine | -4.76 | 7.74E-04 | 3.11 | 2.79E-03 |
| Aspartic acid | -4.61 | 9.62E-04 | 3.02 | 3.25E-03 |
| Lysine | -4.60 | 9.75E-04 | 3.01 | 3.25E-03 |
| Guanosine | -4.55 | 1.06E-03 | 2.98 | 3.40E-03 |
| Aldo/Keto pentose-5-phosphate | -4.51 | 1.13E-03 | 2.95 | 3.51E-03 |
| Gluconic acid | -4.48 | 1.17E-03 | 2.93 | 3.51E-03 |
| Dihydroxyacetone phosphate | -4.31 | 1.54E-03 | 2.81 | 4.46E-03 |
| Adenine | -4.29 | 1.59E-03 | 2.80 | 4.47E-03 |
| Phosphoric acid | -4.23 | 1.75E-03 | 2.76 | 4.68E-03 |
| Creatinine | -4.22 | 1.77E-03 | 2.75 | 4.68E-03 |
| Tetradecanedioic acid | -4.00 | 2.51E-03 | 2.60 | 6.45E-03 |
| Asparagine | -3.71 | 4.04E-03 | 2.39 | 1.01E-02 |
| Glutamic acid | -3.66 | 4.42E-03 | 2.35 | 1.07E-02 |
| Malonic acid | -3.60 | 4.89E-03 | 2.31 | 1.16E-02 |
| Spermidine | -3.46 | 6.08E-03 | 2.22 | 1.40E-02 |
| Succinic acid | -3.40 | 6.75E-03 | 2.17 | 1.52E-02 |
| Lactose | -3.26 | 8.59E-03 | 2.07 | 1.88E-02 |
| Hypotaurine | -2.87 | 1.66E-02 | 1.78 | 3.56E-02 |
| Arachidonic acid | -2.78 | 1.93E-02 | 1.71 | 4.05E-02 |
| Leucine | -2.75 | 2.04E-02 | 1.69 | 4.16E-02 |
| Phenylalanine | -2.74 | 2.08E-02 | 1.68 | 4.16E-02 |
| Pentitols | -2.45 | 3.45E-02 | 1.46 | 6.76E-02 |
| Oleic acid | -2.41 | 3.66E-02 | 1.44 | 6.92E-02 |
| gamma-Tocopherol | -2.41 | 3.69E-02 | 1.43 | 6.92E-02 |
| O-Phosphocolamine | -2.34 | 4.12E-02 | 1.38 | 7.57E-02 |
| Docosanoic acid | -2.31 | 4.34E-02 | 1.36 | 7.81E-02 |
| Methionine sulfoxide | -2.18 | 5.41E-02 | 1.27 | 9.55E-02 |
| Phenol | -2.16 | 5.63E-02 | 1.25 | 9.75E-02 |
Table S5. All significantly different intracellular metabolites identified by t-test analysis incubated with 100 nM 19-OH AD for 3 days.

## Slide 14
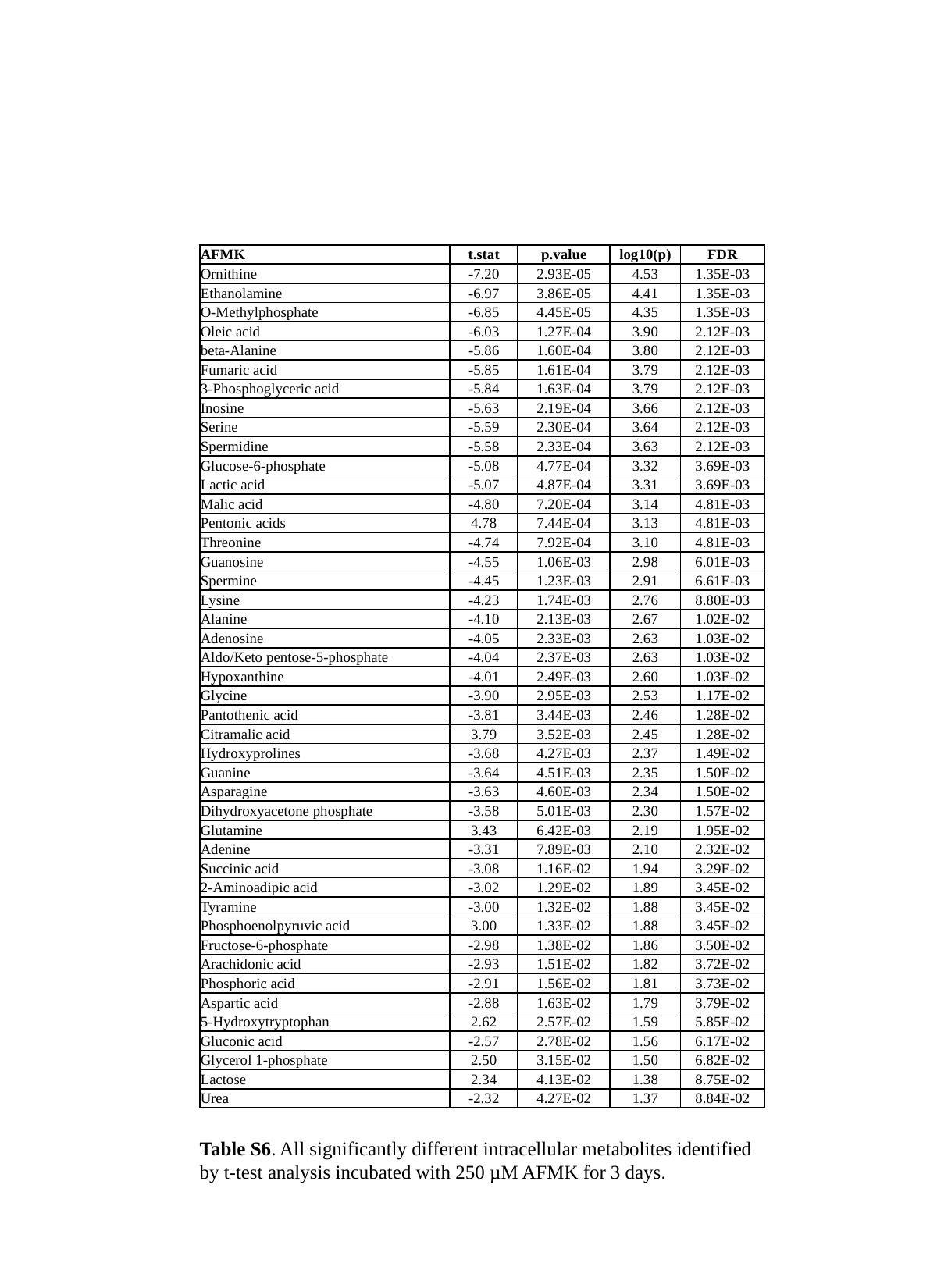

| AFMK | t.stat | p.value | log10(p) | FDR |
| --- | --- | --- | --- | --- |
| Ornithine | -7.20 | 2.93E-05 | 4.53 | 1.35E-03 |
| Ethanolamine | -6.97 | 3.86E-05 | 4.41 | 1.35E-03 |
| O-Methylphosphate | -6.85 | 4.45E-05 | 4.35 | 1.35E-03 |
| Oleic acid | -6.03 | 1.27E-04 | 3.90 | 2.12E-03 |
| beta-Alanine | -5.86 | 1.60E-04 | 3.80 | 2.12E-03 |
| Fumaric acid | -5.85 | 1.61E-04 | 3.79 | 2.12E-03 |
| 3-Phosphoglyceric acid | -5.84 | 1.63E-04 | 3.79 | 2.12E-03 |
| Inosine | -5.63 | 2.19E-04 | 3.66 | 2.12E-03 |
| Serine | -5.59 | 2.30E-04 | 3.64 | 2.12E-03 |
| Spermidine | -5.58 | 2.33E-04 | 3.63 | 2.12E-03 |
| Glucose-6-phosphate | -5.08 | 4.77E-04 | 3.32 | 3.69E-03 |
| Lactic acid | -5.07 | 4.87E-04 | 3.31 | 3.69E-03 |
| Malic acid | -4.80 | 7.20E-04 | 3.14 | 4.81E-03 |
| Pentonic acids | 4.78 | 7.44E-04 | 3.13 | 4.81E-03 |
| Threonine | -4.74 | 7.92E-04 | 3.10 | 4.81E-03 |
| Guanosine | -4.55 | 1.06E-03 | 2.98 | 6.01E-03 |
| Spermine | -4.45 | 1.23E-03 | 2.91 | 6.61E-03 |
| Lysine | -4.23 | 1.74E-03 | 2.76 | 8.80E-03 |
| Alanine | -4.10 | 2.13E-03 | 2.67 | 1.02E-02 |
| Adenosine | -4.05 | 2.33E-03 | 2.63 | 1.03E-02 |
| Aldo/Keto pentose-5-phosphate | -4.04 | 2.37E-03 | 2.63 | 1.03E-02 |
| Hypoxanthine | -4.01 | 2.49E-03 | 2.60 | 1.03E-02 |
| Glycine | -3.90 | 2.95E-03 | 2.53 | 1.17E-02 |
| Pantothenic acid | -3.81 | 3.44E-03 | 2.46 | 1.28E-02 |
| Citramalic acid | 3.79 | 3.52E-03 | 2.45 | 1.28E-02 |
| Hydroxyprolines | -3.68 | 4.27E-03 | 2.37 | 1.49E-02 |
| Guanine | -3.64 | 4.51E-03 | 2.35 | 1.50E-02 |
| Asparagine | -3.63 | 4.60E-03 | 2.34 | 1.50E-02 |
| Dihydroxyacetone phosphate | -3.58 | 5.01E-03 | 2.30 | 1.57E-02 |
| Glutamine | 3.43 | 6.42E-03 | 2.19 | 1.95E-02 |
| Adenine | -3.31 | 7.89E-03 | 2.10 | 2.32E-02 |
| Succinic acid | -3.08 | 1.16E-02 | 1.94 | 3.29E-02 |
| 2-Aminoadipic acid | -3.02 | 1.29E-02 | 1.89 | 3.45E-02 |
| Tyramine | -3.00 | 1.32E-02 | 1.88 | 3.45E-02 |
| Phosphoenolpyruvic acid | 3.00 | 1.33E-02 | 1.88 | 3.45E-02 |
| Fructose-6-phosphate | -2.98 | 1.38E-02 | 1.86 | 3.50E-02 |
| Arachidonic acid | -2.93 | 1.51E-02 | 1.82 | 3.72E-02 |
| Phosphoric acid | -2.91 | 1.56E-02 | 1.81 | 3.73E-02 |
| Aspartic acid | -2.88 | 1.63E-02 | 1.79 | 3.79E-02 |
| 5-Hydroxytryptophan | 2.62 | 2.57E-02 | 1.59 | 5.85E-02 |
| Gluconic acid | -2.57 | 2.78E-02 | 1.56 | 6.17E-02 |
| Glycerol 1-phosphate | 2.50 | 3.15E-02 | 1.50 | 6.82E-02 |
| Lactose | 2.34 | 4.13E-02 | 1.38 | 8.75E-02 |
| Urea | -2.32 | 4.27E-02 | 1.37 | 8.84E-02 |
Table S6. All significantly different intracellular metabolites identified by t-test analysis incubated with 250 µM AFMK for 3 days.

## Slide 15
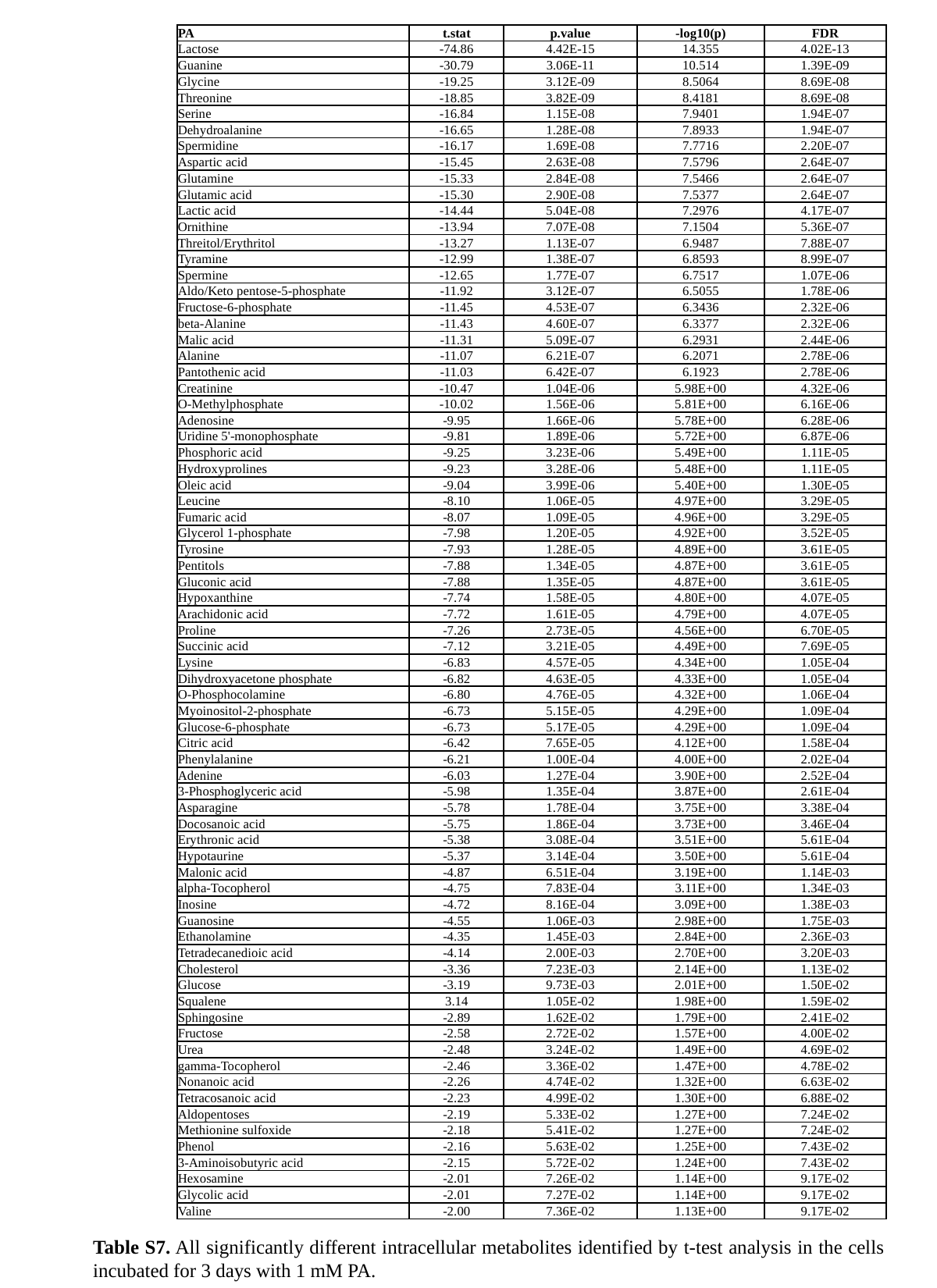

| PA | t.stat | p.value | -log10(p) | FDR |
| --- | --- | --- | --- | --- |
| Lactose | -74.86 | 4.42E-15 | 14.355 | 4.02E-13 |
| Guanine | -30.79 | 3.06E-11 | 10.514 | 1.39E-09 |
| Glycine | -19.25 | 3.12E-09 | 8.5064 | 8.69E-08 |
| Threonine | -18.85 | 3.82E-09 | 8.4181 | 8.69E-08 |
| Serine | -16.84 | 1.15E-08 | 7.9401 | 1.94E-07 |
| Dehydroalanine | -16.65 | 1.28E-08 | 7.8933 | 1.94E-07 |
| Spermidine | -16.17 | 1.69E-08 | 7.7716 | 2.20E-07 |
| Aspartic acid | -15.45 | 2.63E-08 | 7.5796 | 2.64E-07 |
| Glutamine | -15.33 | 2.84E-08 | 7.5466 | 2.64E-07 |
| Glutamic acid | -15.30 | 2.90E-08 | 7.5377 | 2.64E-07 |
| Lactic acid | -14.44 | 5.04E-08 | 7.2976 | 4.17E-07 |
| Ornithine | -13.94 | 7.07E-08 | 7.1504 | 5.36E-07 |
| Threitol/Erythritol | -13.27 | 1.13E-07 | 6.9487 | 7.88E-07 |
| Tyramine | -12.99 | 1.38E-07 | 6.8593 | 8.99E-07 |
| Spermine | -12.65 | 1.77E-07 | 6.7517 | 1.07E-06 |
| Aldo/Keto pentose-5-phosphate | -11.92 | 3.12E-07 | 6.5055 | 1.78E-06 |
| Fructose-6-phosphate | -11.45 | 4.53E-07 | 6.3436 | 2.32E-06 |
| beta-Alanine | -11.43 | 4.60E-07 | 6.3377 | 2.32E-06 |
| Malic acid | -11.31 | 5.09E-07 | 6.2931 | 2.44E-06 |
| Alanine | -11.07 | 6.21E-07 | 6.2071 | 2.78E-06 |
| Pantothenic acid | -11.03 | 6.42E-07 | 6.1923 | 2.78E-06 |
| Creatinine | -10.47 | 1.04E-06 | 5.98E+00 | 4.32E-06 |
| O-Methylphosphate | -10.02 | 1.56E-06 | 5.81E+00 | 6.16E-06 |
| Adenosine | -9.95 | 1.66E-06 | 5.78E+00 | 6.28E-06 |
| Uridine 5'-monophosphate | -9.81 | 1.89E-06 | 5.72E+00 | 6.87E-06 |
| Phosphoric acid | -9.25 | 3.23E-06 | 5.49E+00 | 1.11E-05 |
| Hydroxyprolines | -9.23 | 3.28E-06 | 5.48E+00 | 1.11E-05 |
| Oleic acid | -9.04 | 3.99E-06 | 5.40E+00 | 1.30E-05 |
| Leucine | -8.10 | 1.06E-05 | 4.97E+00 | 3.29E-05 |
| Fumaric acid | -8.07 | 1.09E-05 | 4.96E+00 | 3.29E-05 |
| Glycerol 1-phosphate | -7.98 | 1.20E-05 | 4.92E+00 | 3.52E-05 |
| Tyrosine | -7.93 | 1.28E-05 | 4.89E+00 | 3.61E-05 |
| Pentitols | -7.88 | 1.34E-05 | 4.87E+00 | 3.61E-05 |
| Gluconic acid | -7.88 | 1.35E-05 | 4.87E+00 | 3.61E-05 |
| Hypoxanthine | -7.74 | 1.58E-05 | 4.80E+00 | 4.07E-05 |
| Arachidonic acid | -7.72 | 1.61E-05 | 4.79E+00 | 4.07E-05 |
| Proline | -7.26 | 2.73E-05 | 4.56E+00 | 6.70E-05 |
| Succinic acid | -7.12 | 3.21E-05 | 4.49E+00 | 7.69E-05 |
| Lysine | -6.83 | 4.57E-05 | 4.34E+00 | 1.05E-04 |
| Dihydroxyacetone phosphate | -6.82 | 4.63E-05 | 4.33E+00 | 1.05E-04 |
| O-Phosphocolamine | -6.80 | 4.76E-05 | 4.32E+00 | 1.06E-04 |
| Myoinositol-2-phosphate | -6.73 | 5.15E-05 | 4.29E+00 | 1.09E-04 |
| Glucose-6-phosphate | -6.73 | 5.17E-05 | 4.29E+00 | 1.09E-04 |
| Citric acid | -6.42 | 7.65E-05 | 4.12E+00 | 1.58E-04 |
| Phenylalanine | -6.21 | 1.00E-04 | 4.00E+00 | 2.02E-04 |
| Adenine | -6.03 | 1.27E-04 | 3.90E+00 | 2.52E-04 |
| 3-Phosphoglyceric acid | -5.98 | 1.35E-04 | 3.87E+00 | 2.61E-04 |
| Asparagine | -5.78 | 1.78E-04 | 3.75E+00 | 3.38E-04 |
| Docosanoic acid | -5.75 | 1.86E-04 | 3.73E+00 | 3.46E-04 |
| Erythronic acid | -5.38 | 3.08E-04 | 3.51E+00 | 5.61E-04 |
| Hypotaurine | -5.37 | 3.14E-04 | 3.50E+00 | 5.61E-04 |
| Malonic acid | -4.87 | 6.51E-04 | 3.19E+00 | 1.14E-03 |
| alpha-Tocopherol | -4.75 | 7.83E-04 | 3.11E+00 | 1.34E-03 |
| Inosine | -4.72 | 8.16E-04 | 3.09E+00 | 1.38E-03 |
| Guanosine | -4.55 | 1.06E-03 | 2.98E+00 | 1.75E-03 |
| Ethanolamine | -4.35 | 1.45E-03 | 2.84E+00 | 2.36E-03 |
| Tetradecanedioic acid | -4.14 | 2.00E-03 | 2.70E+00 | 3.20E-03 |
| Cholesterol | -3.36 | 7.23E-03 | 2.14E+00 | 1.13E-02 |
| Glucose | -3.19 | 9.73E-03 | 2.01E+00 | 1.50E-02 |
| Squalene | 3.14 | 1.05E-02 | 1.98E+00 | 1.59E-02 |
| Sphingosine | -2.89 | 1.62E-02 | 1.79E+00 | 2.41E-02 |
| Fructose | -2.58 | 2.72E-02 | 1.57E+00 | 4.00E-02 |
| Urea | -2.48 | 3.24E-02 | 1.49E+00 | 4.69E-02 |
| gamma-Tocopherol | -2.46 | 3.36E-02 | 1.47E+00 | 4.78E-02 |
| Nonanoic acid | -2.26 | 4.74E-02 | 1.32E+00 | 6.63E-02 |
| Tetracosanoic acid | -2.23 | 4.99E-02 | 1.30E+00 | 6.88E-02 |
| Aldopentoses | -2.19 | 5.33E-02 | 1.27E+00 | 7.24E-02 |
| Methionine sulfoxide | -2.18 | 5.41E-02 | 1.27E+00 | 7.24E-02 |
| Phenol | -2.16 | 5.63E-02 | 1.25E+00 | 7.43E-02 |
| 3-Aminoisobutyric acid | -2.15 | 5.72E-02 | 1.24E+00 | 7.43E-02 |
| Hexosamine | -2.01 | 7.26E-02 | 1.14E+00 | 9.17E-02 |
| Glycolic acid | -2.01 | 7.27E-02 | 1.14E+00 | 9.17E-02 |
| Valine | -2.00 | 7.36E-02 | 1.13E+00 | 9.17E-02 |
Table S7. All significantly different intracellular metabolites identified by t-test analysis in the cells incubated for 3 days with 1 mM PA.

## Slide 16
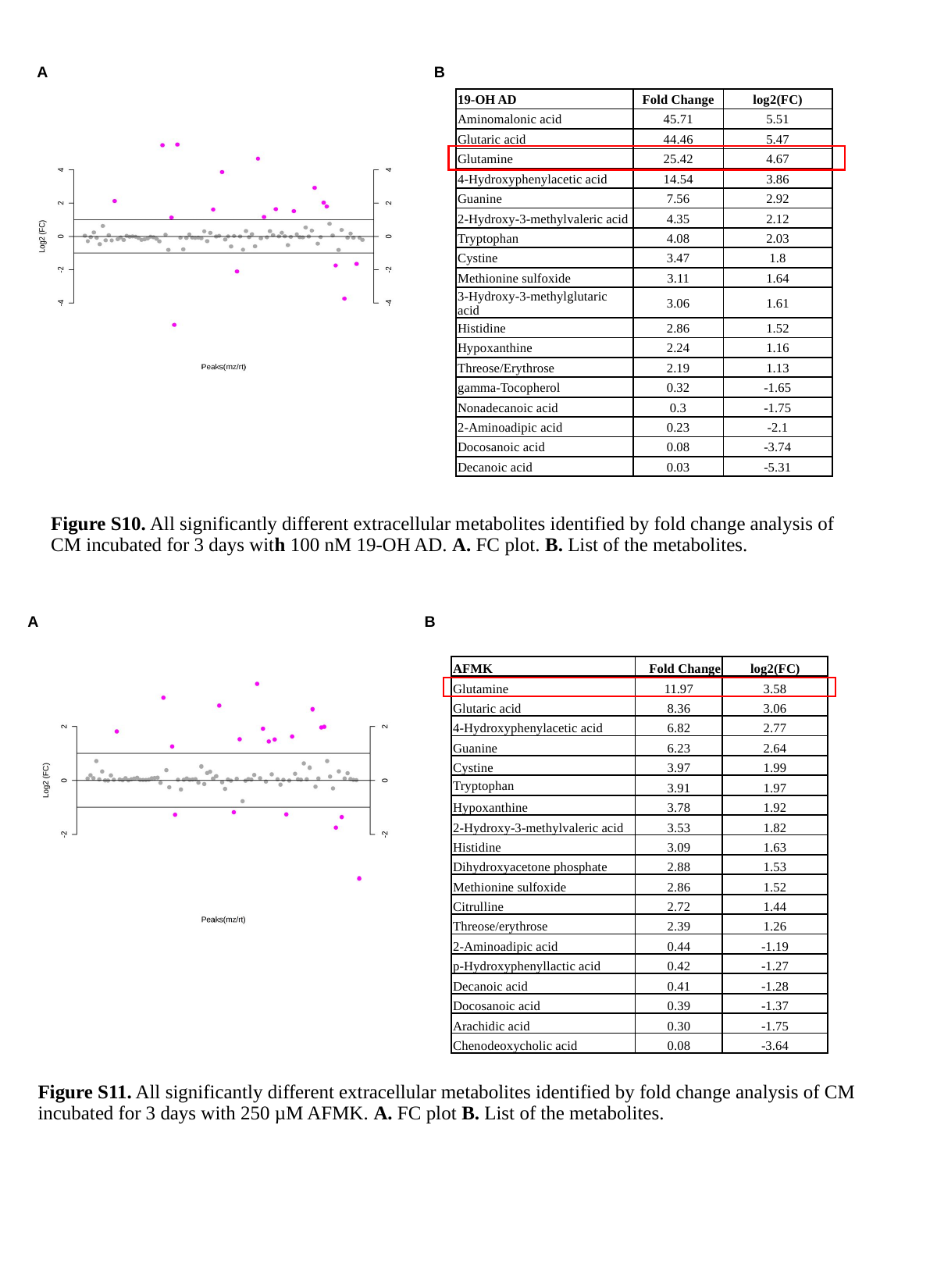

A B
| 19-OH AD | Fold Change | log2(FC) |
| --- | --- | --- |
| Aminomalonic acid | 45.71 | 5.51 |
| Glutaric acid | 44.46 | 5.47 |
| Glutamine | 25.42 | 4.67 |
| 4-Hydroxyphenylacetic acid | 14.54 | 3.86 |
| Guanine | 7.56 | 2.92 |
| 2-Hydroxy-3-methylvaleric acid | 4.35 | 2.12 |
| Tryptophan | 4.08 | 2.03 |
| Cystine | 3.47 | 1.8 |
| Methionine sulfoxide | 3.11 | 1.64 |
| 3-Hydroxy-3-methylglutaric acid | 3.06 | 1.61 |
| Histidine | 2.86 | 1.52 |
| Hypoxanthine | 2.24 | 1.16 |
| Threose/Erythrose | 2.19 | 1.13 |
| gamma-Tocopherol | 0.32 | -1.65 |
| Nonadecanoic acid | 0.3 | -1.75 |
| 2-Aminoadipic acid | 0.23 | -2.1 |
| Docosanoic acid | 0.08 | -3.74 |
| Decanoic acid | 0.03 | -5.31 |
# Figure S10. All significantly different extracellular metabolites identified by fold change analysis of CM incubated for 3 days with 100 nM 19-OH AD. A. FC plot. B. List of the metabolites.
A B
| AFMK | Fold Change | log2(FC) |
| --- | --- | --- |
| Glutamine | 11.97 | 3.58 |
| Glutaric acid | 8.36 | 3.06 |
| 4-Hydroxyphenylacetic acid | 6.82 | 2.77 |
| Guanine | 6.23 | 2.64 |
| Cystine | 3.97 | 1.99 |
| Tryptophan | 3.91 | 1.97 |
| Hypoxanthine | 3.78 | 1.92 |
| 2-Hydroxy-3-methylvaleric acid | 3.53 | 1.82 |
| Histidine | 3.09 | 1.63 |
| Dihydroxyacetone phosphate | 2.88 | 1.53 |
| Methionine sulfoxide | 2.86 | 1.52 |
| Citrulline | 2.72 | 1.44 |
| Threose/erythrose | 2.39 | 1.26 |
| 2-Aminoadipic acid | 0.44 | -1.19 |
| p-Hydroxyphenyllactic acid | 0.42 | -1.27 |
| Decanoic acid | 0.41 | -1.28 |
| Docosanoic acid | 0.39 | -1.37 |
| Arachidic acid | 0.30 | -1.75 |
| Chenodeoxycholic acid | 0.08 | -3.64 |
Figure S11. All significantly different extracellular metabolites identified by fold change analysis of CM incubated for 3 days with 250 µM AFMK. A. FC plot B. List of the metabolites.

## Slide 17
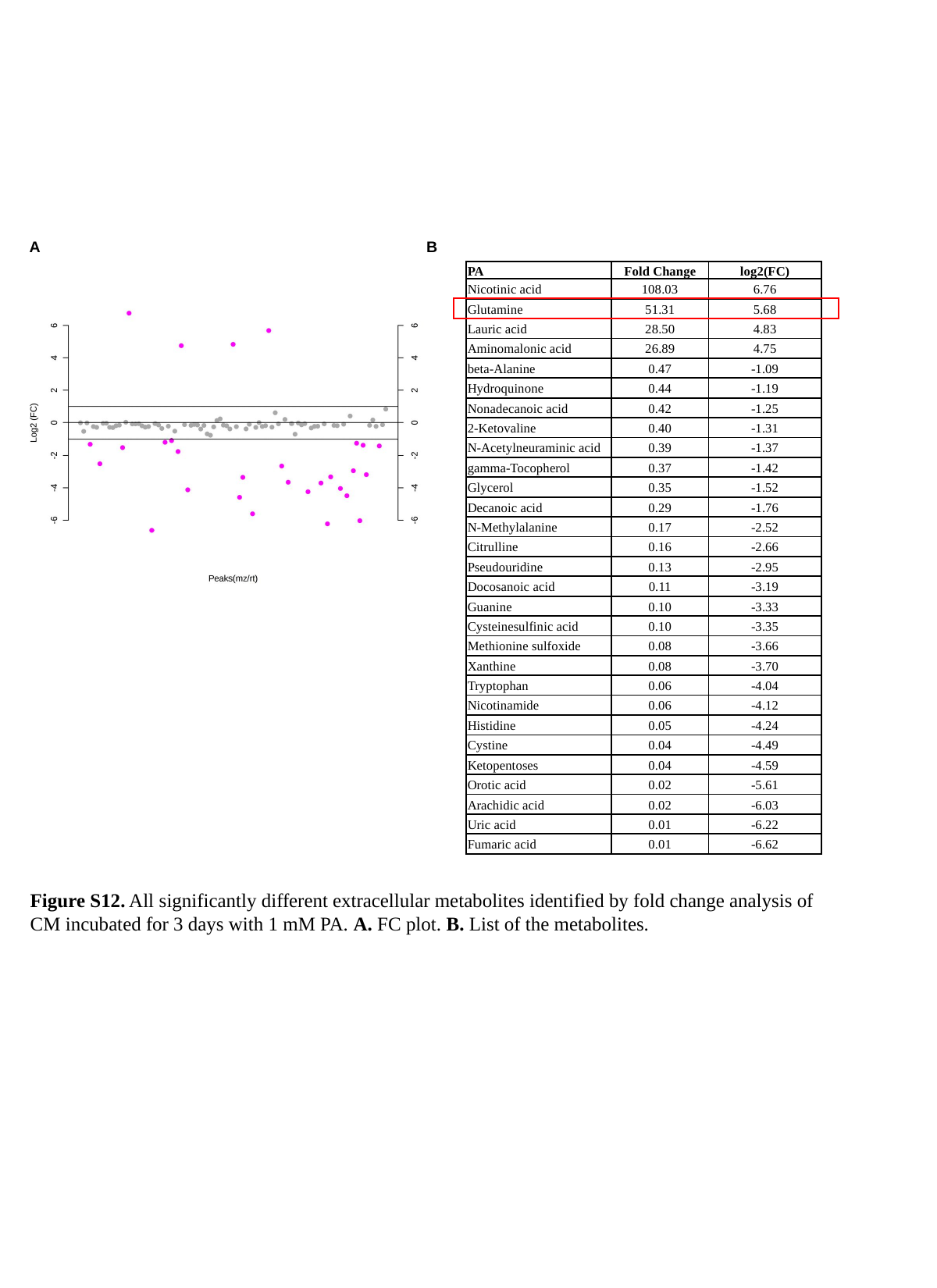

A B
| PA | Fold Change | log2(FC) |
| --- | --- | --- |
| Nicotinic acid | 108.03 | 6.76 |
| Glutamine | 51.31 | 5.68 |
| Lauric acid | 28.50 | 4.83 |
| Aminomalonic acid | 26.89 | 4.75 |
| beta-Alanine | 0.47 | -1.09 |
| Hydroquinone | 0.44 | -1.19 |
| Nonadecanoic acid | 0.42 | -1.25 |
| 2-Ketovaline | 0.40 | -1.31 |
| N-Acetylneuraminic acid | 0.39 | -1.37 |
| gamma-Tocopherol | 0.37 | -1.42 |
| Glycerol | 0.35 | -1.52 |
| Decanoic acid | 0.29 | -1.76 |
| N-Methylalanine | 0.17 | -2.52 |
| Citrulline | 0.16 | -2.66 |
| Pseudouridine | 0.13 | -2.95 |
| Docosanoic acid | 0.11 | -3.19 |
| Guanine | 0.10 | -3.33 |
| Cysteinesulfinic acid | 0.10 | -3.35 |
| Methionine sulfoxide | 0.08 | -3.66 |
| Xanthine | 0.08 | -3.70 |
| Tryptophan | 0.06 | -4.04 |
| Nicotinamide | 0.06 | -4.12 |
| Histidine | 0.05 | -4.24 |
| Cystine | 0.04 | -4.49 |
| Ketopentoses | 0.04 | -4.59 |
| Orotic acid | 0.02 | -5.61 |
| Arachidic acid | 0.02 | -6.03 |
| Uric acid | 0.01 | -6.22 |
| Fumaric acid | 0.01 | -6.62 |
Figure S12. All significantly different extracellular metabolites identified by fold change analysis of CM incubated for 3 days with 1 mM PA. A. FC plot. B. List of the metabolites.

## Slide 18
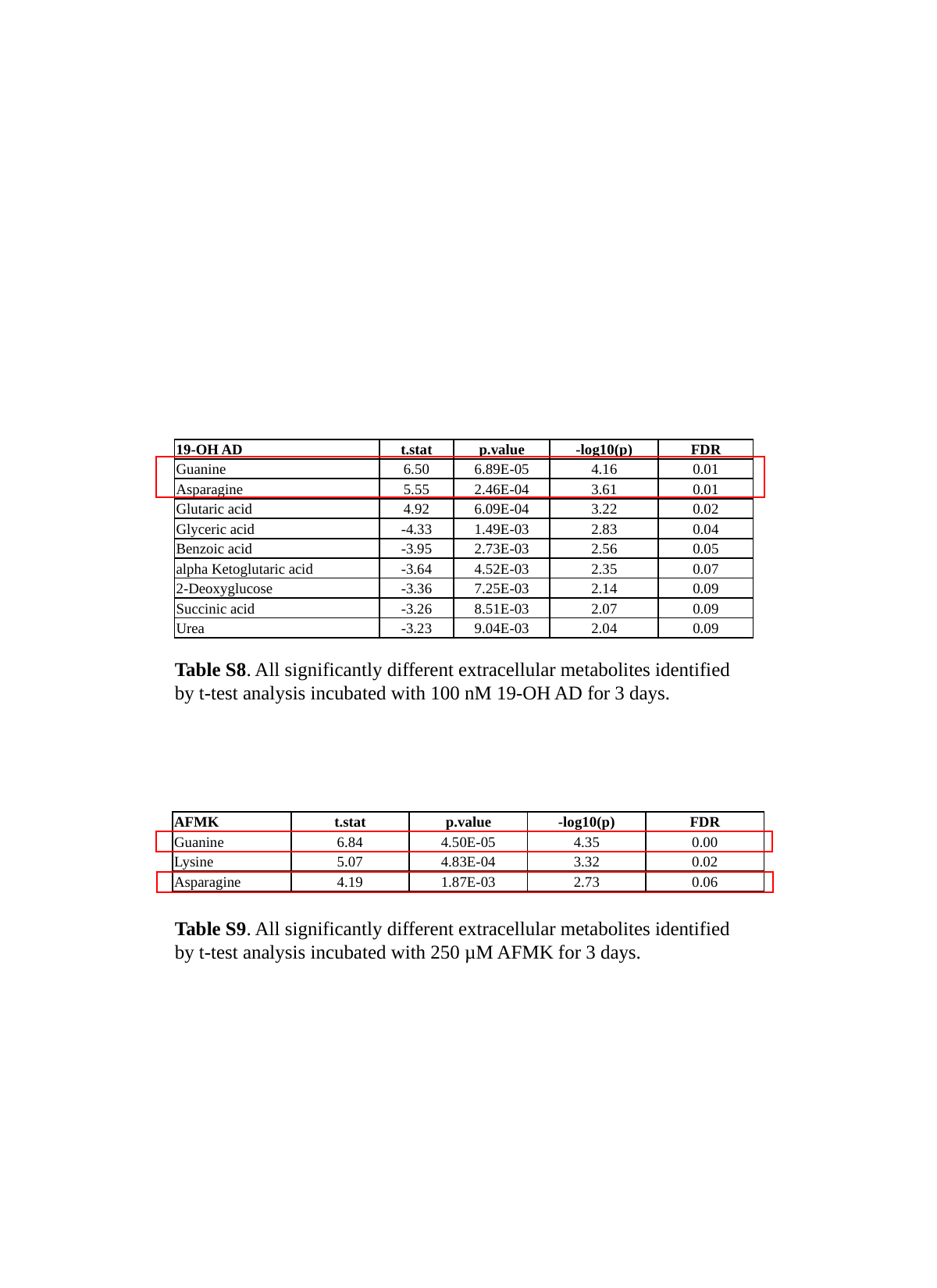

| 19-OH AD | t.stat | p.value | -log10(p) | FDR |
| --- | --- | --- | --- | --- |
| Guanine | 6.50 | 6.89E-05 | 4.16 | 0.01 |
| Asparagine | 5.55 | 2.46E-04 | 3.61 | 0.01 |
| Glutaric acid | 4.92 | 6.09E-04 | 3.22 | 0.02 |
| Glyceric acid | -4.33 | 1.49E-03 | 2.83 | 0.04 |
| Benzoic acid | -3.95 | 2.73E-03 | 2.56 | 0.05 |
| alpha Ketoglutaric acid | -3.64 | 4.52E-03 | 2.35 | 0.07 |
| 2-Deoxyglucose | -3.36 | 7.25E-03 | 2.14 | 0.09 |
| Succinic acid | -3.26 | 8.51E-03 | 2.07 | 0.09 |
| Urea | -3.23 | 9.04E-03 | 2.04 | 0.09 |
Table S8. All significantly different extracellular metabolites identified by t-test analysis incubated with 100 nM 19-OH AD for 3 days.
| AFMK | t.stat | p.value | -log10(p) | FDR |
| --- | --- | --- | --- | --- |
| Guanine | 6.84 | 4.50E-05 | 4.35 | 0.00 |
| Lysine | 5.07 | 4.83E-04 | 3.32 | 0.02 |
| Asparagine | 4.19 | 1.87E-03 | 2.73 | 0.06 |
Table S9. All significantly different extracellular metabolites identified by t-test analysis incubated with 250 µM AFMK for 3 days.

## Slide 19
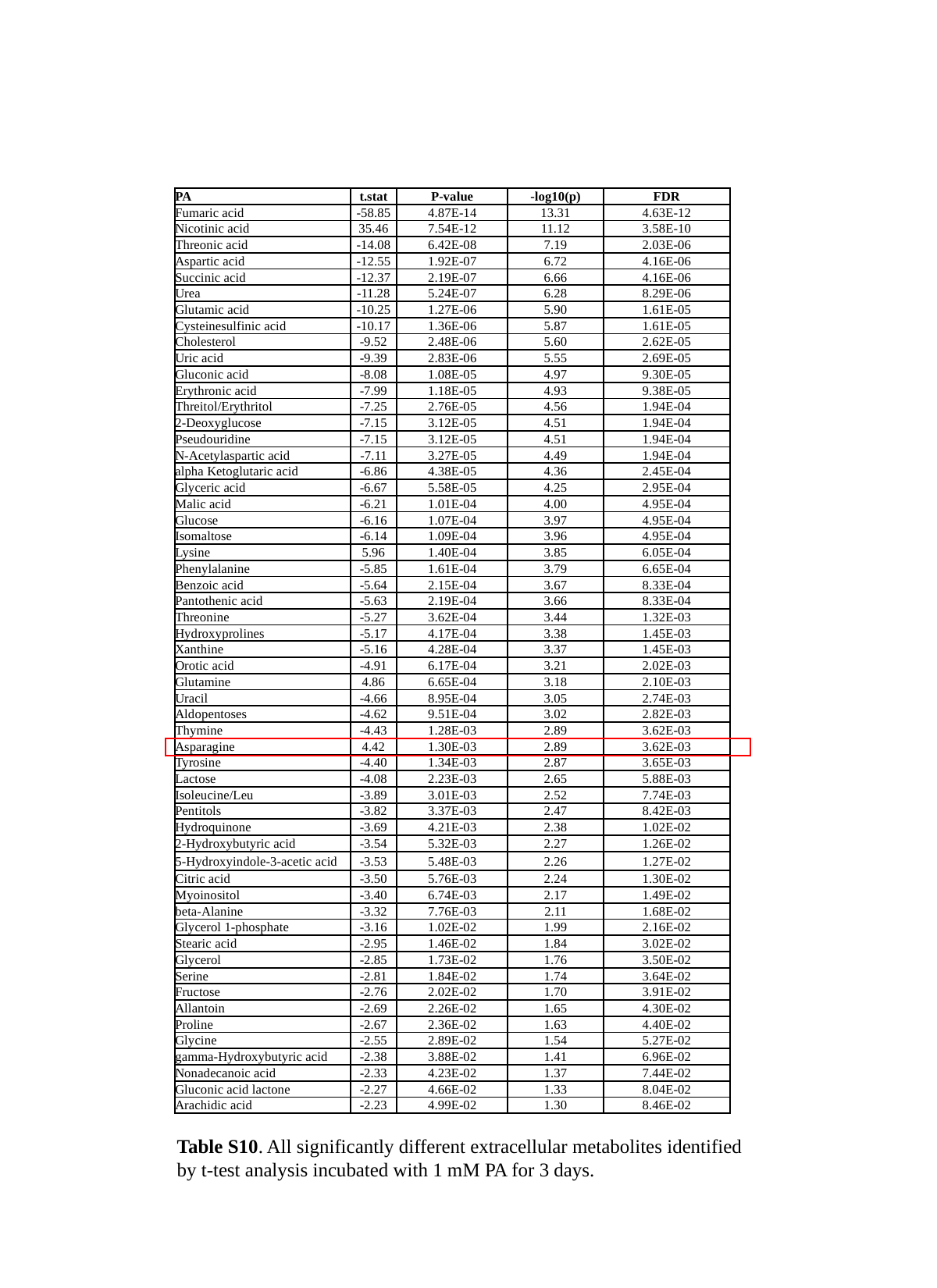

| PA | t.stat | P-value | -log10(p) | FDR |
| --- | --- | --- | --- | --- |
| Fumaric acid | -58.85 | 4.87E-14 | 13.31 | 4.63E-12 |
| Nicotinic acid | 35.46 | 7.54E-12 | 11.12 | 3.58E-10 |
| Threonic acid | -14.08 | 6.42E-08 | 7.19 | 2.03E-06 |
| Aspartic acid | -12.55 | 1.92E-07 | 6.72 | 4.16E-06 |
| Succinic acid | -12.37 | 2.19E-07 | 6.66 | 4.16E-06 |
| Urea | -11.28 | 5.24E-07 | 6.28 | 8.29E-06 |
| Glutamic acid | -10.25 | 1.27E-06 | 5.90 | 1.61E-05 |
| Cysteinesulfinic acid | -10.17 | 1.36E-06 | 5.87 | 1.61E-05 |
| Cholesterol | -9.52 | 2.48E-06 | 5.60 | 2.62E-05 |
| Uric acid | -9.39 | 2.83E-06 | 5.55 | 2.69E-05 |
| Gluconic acid | -8.08 | 1.08E-05 | 4.97 | 9.30E-05 |
| Erythronic acid | -7.99 | 1.18E-05 | 4.93 | 9.38E-05 |
| Threitol/Erythritol | -7.25 | 2.76E-05 | 4.56 | 1.94E-04 |
| 2-Deoxyglucose | -7.15 | 3.12E-05 | 4.51 | 1.94E-04 |
| Pseudouridine | -7.15 | 3.12E-05 | 4.51 | 1.94E-04 |
| N-Acetylaspartic acid | -7.11 | 3.27E-05 | 4.49 | 1.94E-04 |
| alpha Ketoglutaric acid | -6.86 | 4.38E-05 | 4.36 | 2.45E-04 |
| Glyceric acid | -6.67 | 5.58E-05 | 4.25 | 2.95E-04 |
| Malic acid | -6.21 | 1.01E-04 | 4.00 | 4.95E-04 |
| Glucose | -6.16 | 1.07E-04 | 3.97 | 4.95E-04 |
| Isomaltose | -6.14 | 1.09E-04 | 3.96 | 4.95E-04 |
| Lysine | 5.96 | 1.40E-04 | 3.85 | 6.05E-04 |
| Phenylalanine | -5.85 | 1.61E-04 | 3.79 | 6.65E-04 |
| Benzoic acid | -5.64 | 2.15E-04 | 3.67 | 8.33E-04 |
| Pantothenic acid | -5.63 | 2.19E-04 | 3.66 | 8.33E-04 |
| Threonine | -5.27 | 3.62E-04 | 3.44 | 1.32E-03 |
| Hydroxyprolines | -5.17 | 4.17E-04 | 3.38 | 1.45E-03 |
| Xanthine | -5.16 | 4.28E-04 | 3.37 | 1.45E-03 |
| Orotic acid | -4.91 | 6.17E-04 | 3.21 | 2.02E-03 |
| Glutamine | 4.86 | 6.65E-04 | 3.18 | 2.10E-03 |
| Uracil | -4.66 | 8.95E-04 | 3.05 | 2.74E-03 |
| Aldopentoses | -4.62 | 9.51E-04 | 3.02 | 2.82E-03 |
| Thymine | -4.43 | 1.28E-03 | 2.89 | 3.62E-03 |
| Asparagine | 4.42 | 1.30E-03 | 2.89 | 3.62E-03 |
| Tyrosine | -4.40 | 1.34E-03 | 2.87 | 3.65E-03 |
| Lactose | -4.08 | 2.23E-03 | 2.65 | 5.88E-03 |
| Isoleucine/Leu | -3.89 | 3.01E-03 | 2.52 | 7.74E-03 |
| Pentitols | -3.82 | 3.37E-03 | 2.47 | 8.42E-03 |
| Hydroquinone | -3.69 | 4.21E-03 | 2.38 | 1.02E-02 |
| 2-Hydroxybutyric acid | -3.54 | 5.32E-03 | 2.27 | 1.26E-02 |
| 5-Hydroxyindole-3-acetic acid | -3.53 | 5.48E-03 | 2.26 | 1.27E-02 |
| Citric acid | -3.50 | 5.76E-03 | 2.24 | 1.30E-02 |
| Myoinositol | -3.40 | 6.74E-03 | 2.17 | 1.49E-02 |
| beta-Alanine | -3.32 | 7.76E-03 | 2.11 | 1.68E-02 |
| Glycerol 1-phosphate | -3.16 | 1.02E-02 | 1.99 | 2.16E-02 |
| Stearic acid | -2.95 | 1.46E-02 | 1.84 | 3.02E-02 |
| Glycerol | -2.85 | 1.73E-02 | 1.76 | 3.50E-02 |
| Serine | -2.81 | 1.84E-02 | 1.74 | 3.64E-02 |
| Fructose | -2.76 | 2.02E-02 | 1.70 | 3.91E-02 |
| Allantoin | -2.69 | 2.26E-02 | 1.65 | 4.30E-02 |
| Proline | -2.67 | 2.36E-02 | 1.63 | 4.40E-02 |
| Glycine | -2.55 | 2.89E-02 | 1.54 | 5.27E-02 |
| gamma-Hydroxybutyric acid | -2.38 | 3.88E-02 | 1.41 | 6.96E-02 |
| Nonadecanoic acid | -2.33 | 4.23E-02 | 1.37 | 7.44E-02 |
| Gluconic acid lactone | -2.27 | 4.66E-02 | 1.33 | 8.04E-02 |
| Arachidic acid | -2.23 | 4.99E-02 | 1.30 | 8.46E-02 |
Table S10. All significantly different extracellular metabolites identified by t-test analysis incubated with 1 mM PA for 3 days.

## Slide 20
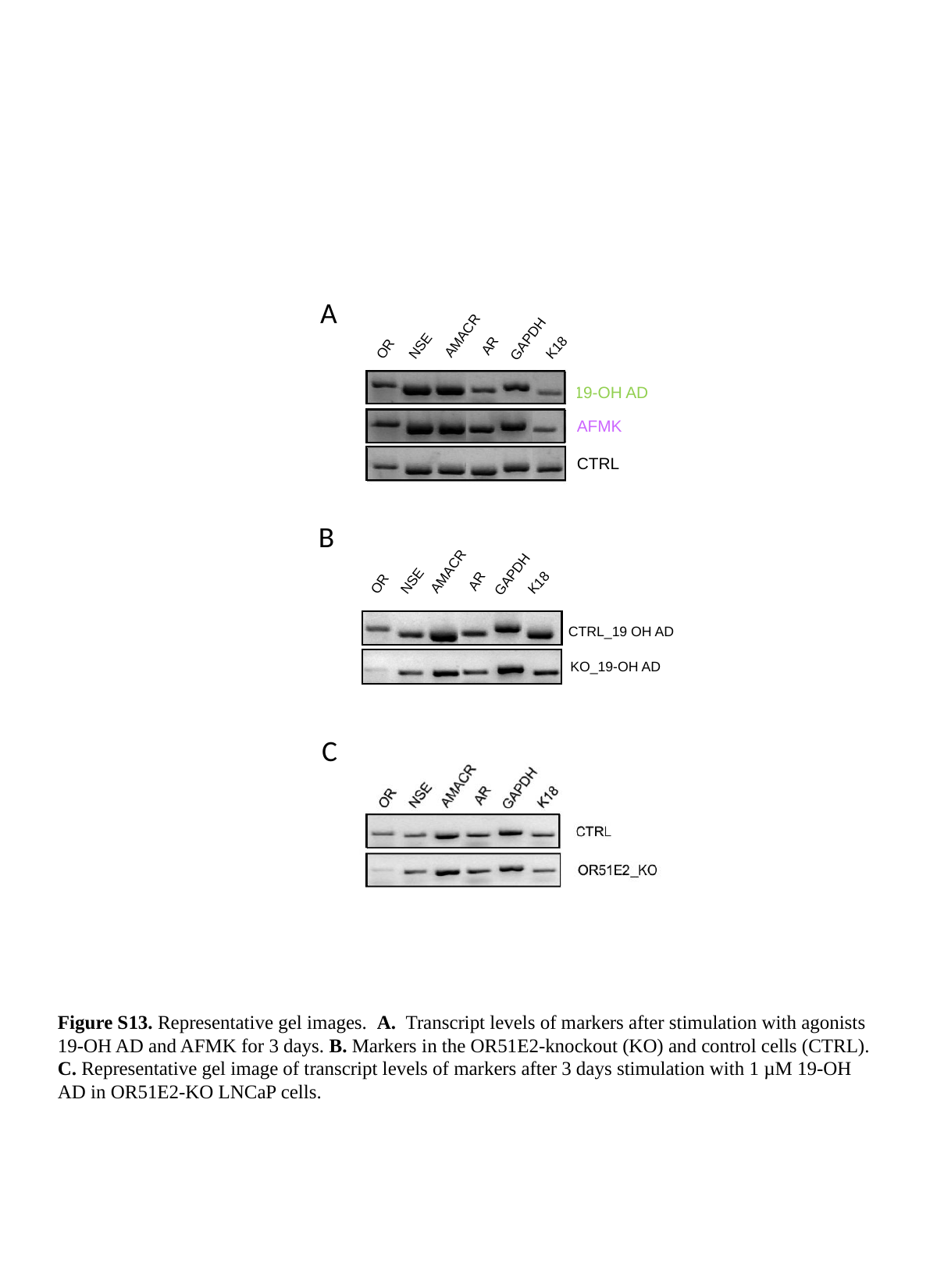

A
AMACR
GAPDH
AR
NSE
K18
OR
19-OH AD
AFMK
CTRL
B
AMACR
GAPDH
AR
NSE
K18
OR
CTRL_19 OH AD
KO_19-OH AD
C
Figure S13. Representative gel images. A. Transcript levels of markers after stimulation with agonists 19-OH AD and AFMK for 3 days. B. Markers in the OR51E2-knockout (KO) and control cells (CTRL). C. Representative gel image of transcript levels of markers after 3 days stimulation with 1 µM 19-OH AD in OR51E2-KO LNCaP cells.
