# Supplementary material for: A Testosterone Metabolite 19-Hydroxyandrostenedione Induces Neuroendocrine Trans-Differentiation of Prostate Cancer Cells via an Ectopic Olfactory Receptor
Source: Front Oncol. 2018 May 28;8:162. doi: 10.3389/fonc.2018.00162 (PMC5985834; doi:10.3389/fonc.2018.00162)
Supplement: Supplementary file 2 [file table_11.docx]

| REAGENT | SOURCE | IDENTIFIER/Cat. No |
| --- | --- | --- |
| Alpha-n-phenylacetyl-l-glutamine | Sigma-Aldrich | CH6371381881 |
| Estriol | Sigma-Aldrich | 285803 |
| Epitestosterone | Sigma-Aldrich | E-058 |
| 19-hydroxyandrost-4-ene-3,17-dione | Sigma-Aldrich | 779717 |
| Testosterone | Steraloids | A6950-000 |
| Palmitic acid | Sigma-Aldrich | P0500 |
| Stearoylethanolamide | Sigma-Aldrich | S8439 |
| Androstenedione | Sigma-Aldrich | A-075 |
| Androstanedione (5alpha androstan 3,17, dione) | Sigma-Aldrich | A-8255 |
| Palmitoylethanolamide | Sigma-Aldrich | P0359 |
| Pentadecanoic acid | Sigma-Aldrich | 91446 |
| Arachidonic acid | Sigma-Aldrich | 10931 |
| 13-cis-retinoic acid | Sigma-Aldrich | R3255 |
| D-alanyl-D-alanine | Sigma-Aldrich | A0912 |
| Glycyl-glycine | Sigma-Aldrich | G0674 |
| 1,4,6 androstatrien-3,17-dione | Steraloids | A4100-000 |
| 6-dehydrotestosterone | Steraloids | A0450-000 |
| Beta-ionone | Sigma-Aldrich | W259500 |
| Kojibiose | Sigma-Aldrich | K4769 |
| Urea | Sigma-Aldrich | 1.08488 |
| N1-acetylspermidine | Bocsci | 14278-49-0 |
| Glyceraldehyde | Sigma-Aldrich | G5001 |
| N8-acetylspermidine | Sigma-Aldrich | A3658 |
| 4-acetamidobutanoic acid | Sigma-Aldrich | S458368 |
| AFMK acetyl-n-formyl-5-methoxykynurenamine | Sigma-Aldrich | A2355 |
| Asymmetric dimethylaariginine | Sigma-Aldrich | COM964465970 |
| Ureidopropionic acid | Sigma-Aldrich | 94295 |
| Ureidoisobutyric acid | Sigma-Aldrich | 74005 |
| N-acetylputrescine | Sigma-Aldrich | A8784 |
| Pelargonidin | Sigma-Aldrich | P1659 |
| Lipoamide | Sigma-Aldrich | T5875 |
| Histamine | Sigma-Aldrich | 53290 |
| Hydroxyacetone | Sigma-Aldrich | 138185 |
| Hydroxypyruvic acid | Sigma-Aldrich | 6372 |
| Imidazolone | Enamine | BBV-44213154 |
| 2-pyrrolidinone | Sigma-Aldrich | 240338 ALDRICH |
| Oxoglutaric acid | Sigma-Aldrich | K1750 |
| L-glyceric acid | Sigma-Aldrich | 51738 |
| Normetanephrine | IsoSciences | ISO-S10416UNL1.0 |
| Glycine | Sigma-Aldrich | 50046 |
| Adenosine 2',3'-cyclic phosphate | Santa Cruz | sc-207262 |
| Gamma-cehc | Cayman-Biomol | CAY-89630 |
| Tetrahydrocurcumin | Santa Cruz | sc-391609 |
| D-maltose | Santa Cruz | sc-215288 |
| Orotidine | Santa Cruz | sc-222103 |
| Acetaminophen glucuronide | BOC Sciences | 16110-10-4 |
| 12,13-epome | Santa Cruz | sc-220590A |
| N-acetylvanilalanine | AKOS | AKOS013465745 |
| Nandrolone | Sigma-Aldrich | N7252 |
| N-acetylglutamic acid | Santa Cruz | 16110-10-4 |
| L-histidinol | AKOS | AKOS006348033 |
| Bradykinin | Sigma-Aldrich | 05-23-0500 |
| Homo-l-arginine | Santa Cruz | sc-479071 |
| Putreanine | AKOS | AKOS005216076 |
| 2,3-diaminopropionic acid | AKOS | AKOS006230079 |
| Akos006230079 | MolPort | MolPort-002-054-631 |

**Table S11**. Chemicals used for in vitro analysis.
